# Pharmacological induction of the hypoxia response pathway in Huh7 hepatoma cells limits proliferation but increases resilience under metabolic stress

**DOI:** 10.1007/s00018-024-05361-6

**Published:** 2024-07-30

**Authors:** Clémence Jacquemin, Walid El Orch, Olivier Diaz, Alexandre Lalande, Anne Aublin-Gex, Florentine Jacolin, Johan Toesca, Mustapha Si-Tahar, Cyrille Mathieu, Vincent Lotteau, Laure Perrin-Cocon, Pierre-Olivier Vidalain

**Affiliations:** 1 CIRI, Centre International de Recherche en Infectiologie, Team Viral Infection, Metabolism and Immunity, Univ Lyon, Inserm, U1111, CNRS, UMR5308, Université Claude Bernard Lyon 1, Ecole Normale Supérieure de Lyon, 69007 Lyon, France; 2 CIRI, Centre International de Recherche en Infectiologie, Team NeuroInvasion, Tropism and Viral Encephalitis, Univ Lyon, Inserm, U1111, CNRS, UMR5308, Université Claude Bernard Lyon 1, Ecole Normale Supérieure de Lyon, 69007 Lyon, France; 3 Centre d’Etude des Pathologies Respiratoires (CEPR), Faculty of Medecine, Inserm, U1100, 37000 Tours, France; 4 Laboratoire P4 INSERM-Jean Mérieux, Lyon, France

**Keywords:** Hepatocellular carcinoma, Cell metabolism, Hypoxia, Mitochondria, HIF-1a, Molidustat

## Abstract

The hypoxia response pathway enables adaptation to oxygen deprivation. It is mediated by hypoxia-inducible factors (HIF), which promote metabolic reprogramming, erythropoiesis, angiogenesis and tissue remodeling. This led to the successful development of HIF-inducing drugs for treating anemia and some of these molecules are now in clinic. However, elevated levels of HIFs are frequently associated with tumor growth, poor prognosis, and drug resistance in various cancers, including hepatocellular carcinoma (HCC). Consequently, there are concerns regarding the recommendation of HIF-inducing drugs in certain clinical situations. Here, we analyzed the effects of two HIF-inducing drugs, Molidustat and Roxadustat, in the well-characterized HCC cell line Huh7. These drugs increased HIF-1α and HIF-2α protein levels which both participate in inducing hypoxia response genes such as BNIP3, SERPINE1, LDHA or EPO. Combined transcriptomics, proteomics and metabolomics showed that Molidustat increased the expression of glycolytic enzymes, while the mitochondrial network was fragmented and cellular respiration decreased. This metabolic remodeling was associated with a reduced proliferation and a lower demand for pyrimidine supply, but an increased ability of cells to convert pyruvate to lactate. This was accompanied by a higher resistance to the inhibition of mitochondrial respiration by antimycin A, a phenotype confirmed in Roxadustat-treated Huh7 cells and Molidustat-treated hepatoblastoma cells (Huh6 and HepG2). Overall, this study shows that HIF-inducing drugs increase the metabolic resilience of liver cancer cells to metabolic stressors, arguing for careful monitoring of patients treated with HIF-inducing drugs, especially when they are at risk of liver cancer.

**Supplementary Information:**

The online version contains supplementary material available at 10.1007/s00018-024-05361-6.

## Introduction

Dioxygen (O_2_) is the final electron acceptor of the mitochondrial respiratory chain and, as such, is essential for the production of ATP and aspartate but also for the reoxidation of ubiquinol (CoQ_10_H_2_) to ubiquinone (CoQ_10_) which is notably required for pyrimidine biosynthesis [1–4]. When O_2_ concentration decreases, the hypoxia response pathway is induced, enabling cells to adapt through metabolic reprogramming [5]. Classically, this results in higher glycolysis coupled with lactate synthesis from pyruvate to maintain ATP level without the need for aerobic respiration. Hypoxia-Inducible Factors (HIFs) are heterodimeric transcription factors playing a key role in the cellular response to hypoxia. HIFs are formed by one alpha subunit (HIF-1α, HIF-2α or HIF-3α) interacting with a beta subunit (HIF-1β). The activation of HIFs in response to O_2_ deprivation is now understood, especially for HIF-1 and HIF-2 that have been extensively studied. At the protein level, Prolyl-Hydroxylase Domain enzymes (PHDs) use O_2_ to hydroxylate two proline residues of HIF-1/2α that are then recognized by the Von Hippel–Lindau (VHL) ubiquitination complex, targeting HIF-1/2α to proteasomal degradation. When O_2_ is missing, HIF-1/2α subunits accumulate and translocate to the nucleus where they bind HIF-1β, forming an active transcription factor that interacts with hypoxia response elements (HREs). Even in normoxic conditions, other molecular mechanisms can also result in the accumulation of HIF-1/2α subunits and activation of the hypoxia response pathway. For example, it was shown in mouse bone marrow-derived macrophages that upon stimulation with bacterial lipopolysaccharide (LPS), ROS production and succinate accumulation induce HIF-1α, which leads to the expression of inflammatory cytokines such as IL-1β [6]. In human monocyte-derived dendritic cells, TLR4 stimulation with LPS induced a p38-MAPK-dependent accumulation of HIF-1α that increased the expression of hexokinase 2 (HK2), the first enzyme of glycolysis [7].

HIF-1 and HIF-2 activate the transcription of more than 200 target genes [8]. These include erythropoietin (EPO) and vascular endothelial growth factor (VEGF), which stimulate erythropoiesis and angiogenesis, respectively. Anemia is a frequent complication in patients with chronic kidney disease (CKD), as the cortical area of their kidneys is often deficient in the production of EPO. This prompted the development of PHD inhibitors (PHDi) such as Molidustat, Daprodustat and Roxadustat which, by inducing HIFs, stimulate EPO secretion in the kidney and liver, thereby increasing erythropoiesis [9]. Other cellular and tissue functions allowing physiological adaptation to hypoxia are also regulated by HIFs, including immune response, cell differentiation, cardiopulmonary function and energy metabolism [10]. By increasing the expression of glucose transporters and glycolytic enzymes, HIFs enhance glucose uptake, glycolytic activity and lactate synthesis to maintain ATP level as mentioned above. In parallel, HIFs induce the expression of Pyruvate Dehydrogenase Kinase 1 (PDK1) to inactivate the Pyruvate Dehydrogenase (PDH) Complex. This inhibits the entry of pyruvate in the Tricarboxylic Acid (TCA) cycle and its conversion to acetyl-CoA, so that lactate synthesis is favored. Protein complexes of the respiratory chain are also remodeled in their composition to limit their activity, mitophagy is induced and mitochondrial biogenesis is inhibited [11].

HIFs also play key roles in many aspects of cancer biology [12]. On the one hand, hypoxia has been shown to interfere with the proliferation of different cell types, including cancer cell lines, and HIF expression has been implicated in cell cycle inhibition through different mechanisms [13]. But on the other hand, HIFs can favor cell adaptation and survival in hypoxic environments [8]. In tumors, HIF expression often contributes to metabolic reprogramming, stem cell specification, epithelial-mesenchymal transition, cell motility and metastasis. It also promotes the expression of autocrine growth factor, tumor vascularization, immune suppression and resistance to treatments [12]. As a result, high levels of HIF expression are generally detrimental and associated to poor prognosis in patients. Interestingly, HIF-1α and HIF-2α protein levels can also be increased in cancer cells due to loss of function of different tumor suppressors, including VHL, which results in either increased HIF-1α synthesis or decreased HIF-1α degradation [14]. Given the role of HIFs in tumor development, drugs that block their transcriptional activity or promote their degradation have been developed and are under clinical evaluation [12, 15]. On the contrary, these observations raised concerns about the carcinogenic potential of PHDi that are used in the treatment of anemia [16]. Preclinical in vivo studies and follow-up of treated patients have been reassuring since no cancer-related adverse events have been established to date [17–21]. Quite surprisingly, experiments performed in vitro or on animal models have even suggested that PHDi could have beneficial effects by inhibiting the growth of some tumors [22–24]. However, these conclusions may not be valid for all types of cancer, or even transposable to patients. Thus, given our limited knowledge of the consequences of long-term treatments with PHDi, patients receiving these drugs should be carefully monitored for tumor development.

The effect of clinically used PHDi on hepatocellular carcinoma (HCC) has not yet been studied, although HCC is the fourth leading cause of cancer-related death, treatments are limited, and its incidence is increasing rapidly [25]. Factors responsible for HCC are quite well characterized and essentially include chronic viral hepatitis, alcohol, mycotoxins and chronic liver inflammation associated to steatohepatitis. HCC is a solid tumor developing hypoxic area as it grows and HIF expression is a prognosis marker associated with poor outcomes [26]. HIFs are also induced after trans-arterial embolization, which is part of HCC treatment procedures, and play a role in tumor resistance to treatment. In standard cell culture conditions in the presence of O_2_, HCC cell lines do not express or express very low levels of HIF-1/2α [27–30], suggesting that HIF induction mostly depends on tumoral micro-environment. To analyze in vitro the effect of clinically used PHDi on HCC, we used the HCC cell line Huh7 which is well-characterized and has a transcription profile highly correlated with HCC tumor samples [31]. To activate the hypoxia response pathway in this cellular model, we used Molidustat [32], a prototypical PHDi that is approved in cats and is in advanced clinical trial in humans for the treatment of CKD-associated anemia. Cellular response to Molidustat was then analyzed by a multi-omics approach including transcriptomics, proteomics and metabolomics together with confocal microscopy. Roxadustat, another PHDi already approved in humans, was also used to validate key results.

## Material and methods

### Cells and reagents

Huh7 (a gift from Dr. Marco Binder; Heidelberg University; Germany) and Huh6 and HepG2 cells (a gift from Pr. Patrice André; Hospices Civils de Lyon; France) were grown in DMEM, high glucose, with GlutaMAX (Ref. 10566016; Gibco; France) supplemented with 10% fetal calf serum (FCS; Biosera; France) and 100 U/mL penicillin/streptomycin (Gibco; France). Cells were grown at 37 °C and 5% CO_2_. Cell lines were authenticated by STR profiling (Cell Line Authentication Service; Eurofins Genomics; Germany), and all cells were tested for mycoplasma contamination (Mycoplasmacheck; Eurofins Genomics; Germany). Molidustat was from Euromedex (France), Antimycin A and Uridine were from Sigma-Aldrich (France), Dipyridamole was from Bertin Bioreagent (France), and GSK2837808A was from MedChemExpress (Sweden). IPPA17-A04 and BAY-2402234 were provided by Yves Janin (Muséum National d’Histoire Naturelle; France).

### Gene expression analysis by RT-qPCR

0.3 × 10^6^ Huh7 cells were seeded in 6-well plates in 3 mL of culture medium. After incubation for 24 h, cells were incubated with fresh culture medium containing Molidustat at 12.5, 25 or 50 μM or DMSO alone. After incubation for 48 h, cells were washed with PBS and processed with the Monarch Total RNA Miniprep Kit (New England BioLabs; France) or the Direct-zol RNA MiniPrep w/Zymo-Spin IIC Columns (Ozyme; France). After quantification, reverse transcription was performed on 400 ng of total RNA with the High-Capacity RNA-to-cDNA Kit (Ref. 4387406; Applied Biosystems; France) following manufacturer’s recommendations. The qPCR was performed with the PowerTrack SYBR Green Master Mix (A46012; Applied Biosystems) on a CFX96 Real-Time PCR Detection System (BioRad; France). Gene induction was expressed a fold change using the 2^−ΔΔCt^ method and RPL13A as a housekeeping gene.

To silence HIF-1α or HIF-2α expression, 0.2 × 10^6^ Huh7 cells were seeded in 6-well plates in 3 mL of culture medium. After 24 h of incubation, supernatant was removed before adding fresh medium without antibiotics. Cells were then transfected with ON-TARGETplus Human HIF-1α siRNA (L-004018-00), HIF-2α siRNA (L-004814-00) or Non-targeting Control Pool control siRNA (D-001810-10) from Horizon Discovery (UK). Cells were transfected with 50 pmol of each siRNA per well and DharmaFECT Transfection Reagent (Horizon Discovery; UK) following manufacturer’s guidelines. After 4 h of incubation, cells were treated with Molidustat (25 μM) or DMSO alone. After 48 h of culture, cells were harvested and processed as described above (Table 1).

**Table 1:** Primers used for RT-qPCR

Name	Forward primer	Reverse primer
BNIP3	CCTTCCATCTCTGCTGCTCTC	AATCCACTAACGAACCAAGTCAG
SERPINE1	CGCTGTCAAGAAGACCCACA	ACCTGCTGAAACACCCTCAC
RPL13A	AAAAGCGGATGGTGGTTCCT	GCTGTCACTGCCTGGTACTT
HIF-1α	CCCATTTTCTACTCAGGACACAG	GCTTGCGGAACTGCTTTCTA
HIF-2α/EPAS1	CTGTGTCTGAGAAGAGTAACTTCC	TTGCCATAGGCTGAGGACTCCT
LDHA	GATTCCAGTGTGCCTGTATGG	TCAGTCCCTAAATCTGGGTGC
EPO	AGAAGGAAGCCATCTCCCCT	TACAGCTTCAGCTTTCCCCG

### Analysis of protein expression by Jess and western-blot

0.5 × 10^6^ Huh7 cells were seeded in 6-well plates with 3 mL of culture medium containing Molidustat at 25 μM or DMSO alone. After incubation for 24 h, cells were washed with PBS and stored at − 20 °C. Cells were lysed on ice for 20 min with 300 μL of RIPA lysis buffer (20-188; Millipore; France) supplemented with Protease Cocktail Inhibitor (P8340; Sigma-Aldrich). Lysates were collected and clarified by centrifugation at 4 °C (20 min; 17,000 g). After protein quantification (DC Protein Assay; Bio-Rad), proteins extracts were analyzed for HIF-1α and HIF-2α expression with the Jess Simple Western System from Bio-Techne following manufacturer’s recommendations (12–230 kDa Fluorescence Separation Module, SM-FL001; EZ Standard Pack 1; PS-ST01EZ; Total Protein Detection Module for Chemiluminescence based total protein assays, DM-TP01; RePlex™ Module, RP-001). Protein extracts were dispensed in the assay plate at a final concentration of 0.2 μg/μL. HIF-1α and HIF-2α (respectively NB100-134 and NB100-122; 1/100 final dilution; Novus Biologicals; France) were detected with an anti-rabbit polyclonal antibody (Anti-Rabbit Detection Module; DM-001; Bio-Techne).

For detecting VDAC expression, cellular lysates were analyzed by Western blotting using NuPAGE 4 to 12%, Bis–Tris, 1.5 mm, Mini Protein Gels (NP0335; Invitrogen; France). Proteins were transferred on Nitrocellulose membranes with the Trans-Blot Turbo Transfer System (Ref. 1704271; 25 V; 1.3A; 10 min; BioRad). Membranes were blocked in PBST plus 5% dry nonfat milk, and VDAC expression was detected using a mouse monoclonal antibody (MABN504; clone N152B/23; 1/500 dilution; Sigma-Aldrich). The HRP-conjugated, goat anti-mouse IgG antibody from Jackson ImmunoResearch (115-036-003) was used at a 1/1000 dilution. HRP activity was detected with the SuperSignal West Pico PLUS Chemiluminescent Substrate (Ref. 34578; Thermo Scientific; France).

### Proteomic analysis

Six biological replicates were prepared for each culture condition. Three million Huh7 cells were grown in 10 mL of culture medium with Molidustat at 25 μM or DMSO alone in 10 cm dishes for 48 h. Cells were washed with ice-cold PBS, harvested by scrapping and washed again with PBS after centrifugation. Frozen cellular pellets stored at − 80 °C were shipped for analysis to Doppleganger Biosystem GmbH (Germany) and processed as previously described by positive-pressure filter-aided sample preparation in 96-well format [33]. After trypsin digestion, LC–MS was performed on a U3000 RSLCnano ProFlow system coupled to a Q Exactive HF mass spectrometer (Thermo Fisher Scientific). Raw data were processed as previously described [33] with the Proteome Discoverer platform 2.3 together with Percolator and Peptide validator to adjust the false discovery rate to 1%. Signal intensities were obtained for each protein and the complete dataset is available in Table S1. The mass spectrometry proteomics data have been deposited to the ProteomeXchange Consortium via the PRIDE partner repository with the dataset identifier PXD049964 [34].

### Transcriptomic analysis

Three biological replicates were prepared for each culture condition. 0.4 × 10^6^ Huh7 cells were seeded in 6-well plates and grown in 2 mL of culture medium with Molidustat (25 μM) or DMSO alone for 24 or 48 h. To collect samples, cells were washed with ice-cold PBS and lysed in 350 μL of RLT lysis buffer (Qiagen; France). Frozen samples were stored at − 80 °C before shipping to Viroscan3D (France). Total RNA was extracted using the RNeasy Mini kit including DNAse treatment (Qiagen). Samples were quantified and qualified using SS RNA System on Fragment Analyzer (Agilent; USA). Transcriptome profiling was performed by high-throughput 3’ UTR RNA sequencing of polyadenylated RNAs. The QIAseq UPX 3' Transcriptome Kit (Qiagen) was used for reverse transcription and to generate cDNA library. Then, gene expression was analyzed by Next-Generation Sequencing (NGS) using Illumina NextSeq 500 (Paired end reads). Reads were mapped on the reference genome Homo sapiens (GRCh38). Raw data were processed using STAR 2.7.9a for mapping reads on human genome, HTSeq 0.11.2 for counting tags and the DESeq2 R package (v1.26.0) pipeline to normalize data and identify differentially expressed genes. Raw data are available on the Gene Expression Omnibus database (Accession number GSE242340). Molidustat was considered to have a significant effect on gene expression when adjusted p-value was below 0.05. The Gene Set Enrichment Analysis (GSEA) was performed as previously described [35].

### Metabolomic analysis

Four biological replicates were prepared for each culture condition. 2 × 10^6^ Huh7 cells were seeded in 10 mm dishes and grown for 24 h in 10 mL of culture medium. Supernatant was removed and replaced by 8 mL of fresh culture medium containing DMSO alone, Molidustat (25 µM), IPPA17-04 (2 µM) or BAY-2402234 (2 µM) for 24 h. Cells medium was removed and metabolites were immediately extracted in 2 mL ice-cold (− 20 °C) 80% MS-grade MeOH (Ref. 900688; Sigma-Aldrich) in sterile pyrogen free water Otec (Ref. 600500; Aguettant; France). After vortexing for 1 min, samples were stored at − 80 °C before shipping in dry ice. Sample analysis was carried out by MS-Omics (Denmark) as follows. Samples were dried under nitrogen flow and reconstituted in 140 μl MQW. After reconstitution samples were filtered and additionally diluted 10 times in eluent A for semi-polar metabolites analysis and 5 times in eluent A for polar metabolites analysis. The analyses were carried out using a Thermo Scientific Vanquish LC coupled with a high-resolution quadrupole-orbitrap mass spectrometer (Q Exactive^™^ HF Hybrid Quadrupole-Orbitrap, Thermo Fisher Scientific). An electrospray ionization interface was used as ionization source. Analysis was performed in negative and positive ionization mode. For semi-polar metabolites, the UPLC was performed using a slightly modified version of the protocol described by C.E. Doneanu et al. [36]. For polar metabolites, the UPLC was performed using a slightly modified version of the protocol described by Hsiao et al. [37]. Peak areas were extracted using Compound Discoverer 3.2 (Thermo Fisher Scientific) and Skyline [38]. Identification of compounds were performed at four levels; Level 1: identification by retention times (compared against in-house authentic standards), accurate mass (with an accepted deviation of 3 ppm), and MS/MS spectra, Level 2a: identification by retention times (compared against in-house authentic standards), accurate mass (with an accepted deviation of 3 ppm). Level 2b: identification by accurate mass (with an accepted deviation of 3 ppm), and MS/MS spectra, Level 3: identification by accurate mass alone (with an accepted deviation of 3 ppm). A total of 2115 compounds were detected in the samples. Hereof, were 405 annotated on level 3, 146 on level 2b, 84 on level 2a, and 110 on level 1. Compounds analyzed in this study are from Level 1 or 2a. The results from the analyses are presented as log2 values of the ratio between the average of Molidustat vs DMSO treated samples (Supplementary Fig. 2A; n = 4).

### Lactate quantification

Secreted lactate was measured with a commercial assay (Lactate Assay Kit MAK064, Sigma-Aldrich). Briefly, supernatants of Huh7 cells were collected and filtered through Amicon Ultra-0.5 Centrifugal Filter Units with a 10 kDa cut-off (UFC5010, Millipore) for 15 min at 13,000 g and 4 °C. Deproteinized samples were diluted 1:50 in assay buffer. Lactate quantity was assessed according to the manufacturer’s instructions.

### Real-time monitoring of metabolic phenotype with a Seahorse XF analyzer

Cells were seeded in Seahorse XF 24-wells microplates (Agilent), coated with Poly-L-Lysine 0.01% (P4832; Sigma-Aldrich), at 5 × 10^4^ cells/well in 250 µL of DMEM medium (Ref. 31053044; Gibco) supplemented with 10% FCS (Biosera), 2 mM l-glutamine, 100 U/mL penicillin/streptomycin, treated with 25 µM Molidustat or DMSO alone and incubated at 37 °C and 5% CO_2_ for 48 h. The assay was initiated by replacing growth medium with prewarmed Seahorse assay medium (XF DMEM pH7.4; 103575-100; Agilent) supplemented with glucose (10 mM), l-glutamine (2 mM) and sodium pyruvate (1 mM). Cells were washed with 1 mL assay medium and incubated at 37 °C for 1 h without CO_2_. The medium was replaced by 500 µL of prewarmed assay medium prior measurement of oxygen consumption rate (OCR) and extracellular acidification rate (ECAR) with the Seahorse XFe24 analyzer using the MitoStress test assay (Agilent). The number of cells was determined at the end of the run after Hoechst staining and cell counting using Cytation 1 cell imaging reader (Agilent BioTek). Results were normalized by cell count and analyzed using the Seahorse Wave software.

### Fluorescence imaging and quantitative analysis of mitochondria

Cells were seeded in 24-well plates containing poly-L-lysine-coated coverslips at 15 × 10^3^ cells/well in 500 µL of culture medium with Molidustat at 25 μM or DMSO alone. After 48 h of culture, cell culture medium was removed and mitochondria were stained with a MitoTracker Red CMXRos solution in culture medium at 250 nM (M7512; Invitrogen). Cells were incubated for 30 min at 37 °C, washed with fresh culture medium and fixed with 4% formaldehyde. After 15 min of incubation at 37 °C, cells were washed with PBS and nuclei were stained with Hoechst (NucBlue Fixed Cell ReadyProbes; R37606; Invitrogen). After 5 min of incubation at room temperature (RT), coverslips with cells were washed with PBS and mounted for fluorescence microscopy imaging using the ProLong^™^ Diamond Antifade Mountant (P36970; Invitrogen). Cells were then imaged on a Yokogawa HCS CQ1 spinning disk confocal system, using a 40X Olympus UPLSAPO40X2 objective. *Z*-stacks of 7-µm-range with an imaging step of 0.33 µm were acquired in random areas of the slides, with a 561 nm and a 405 nm laser (excitation power 50%) and an exposure time of 500 ms. Images were analyzed as 3D stacks using Fiji (v2.14.0) plugin Mitochondria Analyzer (v2.3.1) ([39], https://github.com/AhsenChaudhry/Mitochondria-Analyzer). Images were first converted to 8-bit format and the scale was set at 0.16 µm/pixel before image processing and thresholding. Mitochondria of individual cells were analyzed by manually cropping the images before using the following pre-processing commands of the "3D Threshold” command: Subtract Background (rolling ball radius = 1 µm), Sigma Filter Plus (radius = 1 µm), Enhance Local Contrast (max slope = 1.25), and Adjust Gamma (gamma = 0.9). The local threshold method was set as “Weighted Mean” with a block size of 1.4 µm and a C-Value of 1.2. Finally, the following post-processing commands were applied: Despeckle, Remove Outliers (outlier radius = 0.9375 pixels), and Fill 3D Holes. The “3D Analysis” command was then used to quantify mitochondria morphological and network characteristics. The optimal parameters were identified using the “3D Threshold Optimize” command by testing different ranges of settings. Thirty cells were analyzed by condition.

### Quantification of cell proliferation

Cell counts in culture wells were determined with the CellTiter-Glo Luminescent Cell Viability Assay (G7570; Promega; France), which relies on ATP quantification as a proxy of the number of cells in a well, or by staining cell nuclei with Hoechst followed by the quantification of the fluorescent signal. Huh7 and HepG2 cells were seeded at 5 × 10^3^ cells/well and Huh6 cells at 2 × 10^3^ cells/well ﻿in triplicates in white 96-well plates for the CellTiter-Glo detection or in sextuplicates in black plates with clear bottoms for fluorescence-based cell quantification. Cells were incubated for 96 h in 200 µL of culture medium with DMSO alone or indicated drugs (Molidustat at 25 µM, IPPA17-A04 at 1 µM, Dipyridamole at 2 µM, Antimycin A at 0.04 µM). For ATP quantification in culture wells, 100 µL of culture medium were removed first, and 50 µL of CellTiter-Glo reagent were added in each well. After 10 min of incubation at RT, luminescence was quantified with Tristar 5 Multimode reader (Berthold; Germany). For staining cell nuclei, Hoechst 33342 (62249; Thermo Scientific) was added to each well at 40 µM and incubated for 30 min at 37 °C. Wells were washed with PBS and for Huh7 and HepG2 cells, mean fluorescence in each well was measured with a Tristar 5 Multimode reader (Berthold; France). For Huh6 cells, fluorescent cells were numbered with a Celigo Image Cytometer (Revvity; France).

### Cytoxoxicity assay

Cytotoxicity was evaluated by CellTox™ Green cytotoxicity assay (G8741, Promega). 5 × 10^3^ cells/well were plated in 96-well plates, and cultured in the presence of CellTox green dye (1/500 final dilution) and treated as indicated. Fluorescence was measured (Ex:490 nm; Em:520 nm) every 24 h using TECAN M200 microplate reader. Treatment with 10% Triton X-100 one hour before measurement was used as cytotoxicity control.

### Real-time cell analysis (RTCA) by impedancemetry

Cellular proliferation was monitored by impedancemetry using the xCELLigence RTCA MP system (Agilent) in a 96-well format (RTCA E-Plates; Ref. 300600890; Agilent). Huh7 cells were seeded at 4 × 10^3^ cells/well in duplicates in 200 µL of culture medium with DMSO alone or Molidustat at 25 µM. Cells were grown at 37 °C and 5% CO_2_, and the Cell Index (CI) was determined every 15 min for 96 h. The area under CI curves was calculated from 0 to 96 h of treatment.

### Measurement of DHODH activity

The protocol to measure DHODH activity in cellular lysates was adapted from Yin et al. [40]. 6 × 10^6^ cells were harvested by trypsinization and washed twice with PBS. The cellular pellet was resuspended in 500 µL of water at 4 °C and sonicated for 1 min. Cellular lysate was centrifuged at 16,000 g for 20 min at 4 °C. Supernatant was recovered to measure DHODH activity. In Eppendorf tubes, 240 µL of cellular lysate were mixed with 80 µL of dihydroorotate solution (5 mM in water; D7128; Sigma-Aldrich), 240 µL of K_2_CO_3_-HCl solution (1 M in water; pH = 8), 280 µL of decylubiquinone solution (1 mM; 3.2% DMSO in water; D7911; Sigma-Aldrich) and 240 µL of water. In control tubes, IPPA17-A04 was added at a final concentration of 2 µM. The tubes were incubated at 37 °C, and 40 µL were harvested every 5 min. Reaction was stopped on ice. For the detection of orotate synthesis, the following reagents were added to each tubes: 60 µL of water, 100 µL of 4-TFMBAO (4 mM in water; 542822; Sigma-Aldrich), 100 µL of K_3_[Fe(CN)_6_] (8 mM in water) and 100 µL of K_2_CO_3_ (80 mM in water). Tubes were incubated for 5 min at 80 °C. 150 µL of each tube were transferred to a black 96-well plate and fluorescence was determined (Ex: 340 nm; Em: 460 nm).

### Bioinformatics and statistical analyses

Venn diagrams were generated with the online tool available at https://bioinformatics.psb.ugent.be/webtools/Venn/. Functional enrichment analyses were performed with DAVID using the “Biological Process” (BP) annotation of the GO database [41, 42]. The list of metabolites up and downregulated by Molidustat was analyzed with MetaboAnalyst 6.0 (Homo sapiens SMPDB pathway library; [63]). All statistical analyses were performed with GraphPad Prism v10. BioRender was used for graphic representations.

## Results

### Molidustat induces the hypoxia response pathway in Huh7 cells

**Fig. 1:**
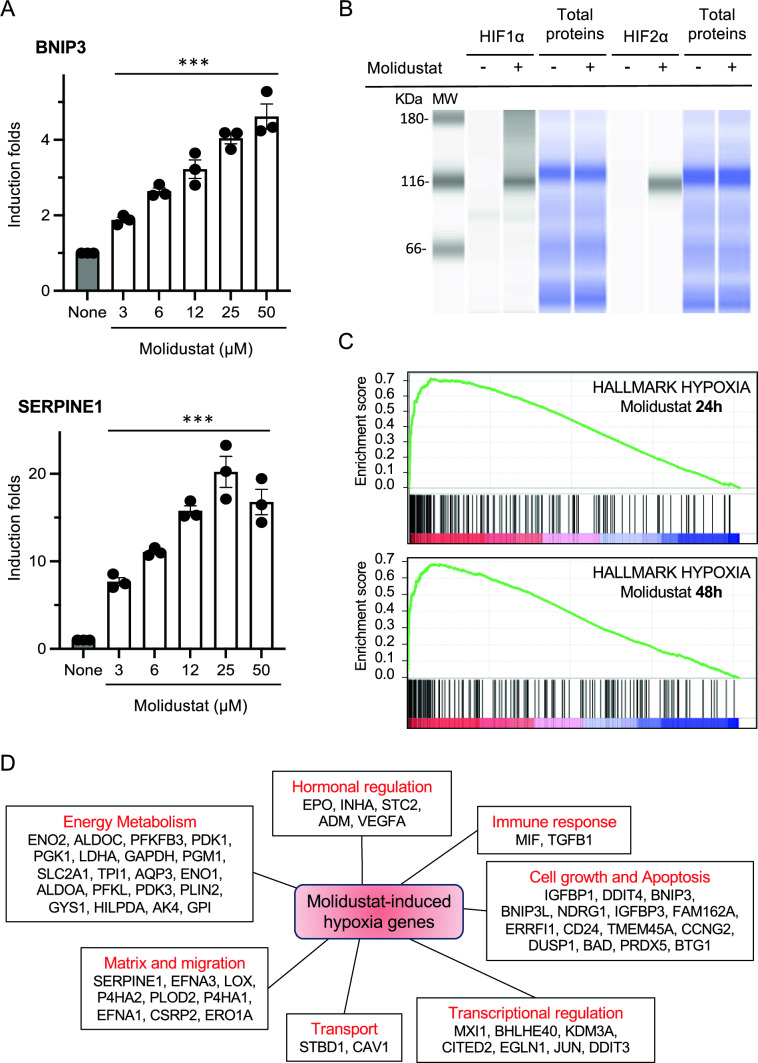
Molidustat induces the hypoxia response pathway in Huh7 cells. **A** Huh7 were stimulated for 48 h with Molidustat at 3, 6, 12.5, 25 or 50 µM or DMSO alone (None). BNIP3 and SERPINE1 expression was determined by RT-qPCR using RPL13A as housekeeping gene. Means ± SEM of three experiments. ***p < 0.001; Friedman test. **B** HIF-1α and HIF-2α are induced at protein level by Molidustat. Huh7 cells were treated for 24 h with Molidustat (25 µM) or DMSO alone, and HIF-1α and HIF-2α expression were determined by Jess analysis. Total proteins loaded in the capillaries are presented on the right panel. **C** GSEA analysis of differentially expressed genes in Huh7 cells treated for 24 h or 48 h with Molidustat (25 µM) or DMSO alone. **D** This figure shows genes upregulated in Molidustat-treated Huh7 cells (Log_2_(FC) > 1 after 24 h or 48 h of treatment; p-adj < 0.05) that are associated to hypoxia response in the molecular signature database MSigDB (Hallmark Hypoxia) or in the Gene Ontology database (GO:0001666). ALKBH5 and WSB1 were not shown as they did not fall into one of the indicated categories

The activation in Huh7 cells of the hypoxia-response pathway by Molidustat was first determined by monitoring with RT-qPCR the expression of BNIP3 and SERPINE1, two marker genes induced by hypoxia in HCC cell lines [43, 44]. As shown in Fig. 1A, these two genes were upregulated when treating Huh7 cells for 48 h with Molidustat. Based on the induction profile of these two genes, the concentration of 25 μM which corresponds to a plateau of activity, and is consistent with previous in vitro studies [23, 45, 46], was selected for subsequent experiments. At this concentration, HIF-1α and HIF2-α protein expression was induced by Molidustat as assessed by automated, capillary-based protein separation and Western blotting (Fig. 1B). In contrast, HIF-1α and HIF2-α mRNA were unaffected by Molidustat (Supplementary Fig. 1A). Overall, this is consistent with the expected regulation of HIF-1/2α at the protein level rather than at the transcriptional level. Roxadustat also increased mRNA levels of BNIP3 and SERPINE1 and induced the accumulation of HIF-1/2α proteins in Huh7 cells (Supplementary Fig. 1B and C). This extends our observations to another PHDi which has a chemical structure different from Molidustat.

To further explore the effect of Molidustat on gene expression, the transcriptome of Huh7 cells was analyzed by next-generation sequencing after 24 h or 48 h of treatment to capture early and late regulatory events. In total, 288 genes were induced by Molidustat after either 24 or 48 h (Table S2) and 906 genes were downregulated (Table S3). Gene Set Enrichment Analysis (GSEA) showed a strong upregulation of the “hypoxia response” gene set at both timepoints (Fig. 1C and Table S4). Other induced gene sets were glycolysis, angiogenesis and epithelial-mesenchymal transition that are all biological functions associated to hypoxia response (Table S4; [47]). Finally, we found that amongst all the genes induced by Molidustat, 21.5% (i.e. 62 genes) were assigned to hypoxia response either in the molecular signature database MSigDB (Hallmark Hypoxia) or in the Gene Ontology database (GO:0001666). Figure 1D highlights the main functions associated to these genes. Altogether, these results demonstrate that Molidustat stimulates the hypoxia response pathway in Huh7 cells as expected for a PHDi.

**Fig. 2:**
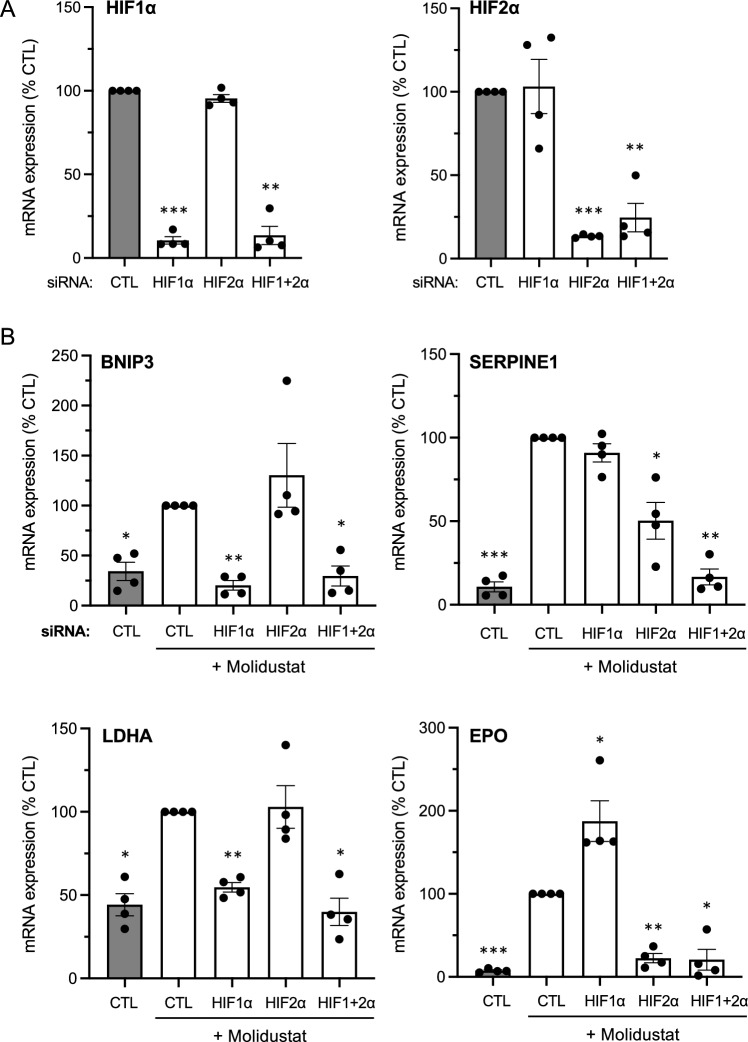
Induction of hypoxia response genes by Molidustat depends both on HIF-1α and HIF-2α. **A** Huh7 cells were transfected with control siRNA (CTL) or siRNA targeting HIF-1α, HIF-2α or both combined. After 48 h of incubation, HIF-1α and HIF-2α expression was determined by RT-qPCR using RPL13A as housekeeping gene. Data were normalized to cells transfected with control siRNA. Means ± SEM of 4 experiments. *p < 0.05, **p < 0.01, ***p < 0.001; one sample t-test using 100% as reference and Holm-Šidák correction for multiple testing. **B** Same experiment as in (**A**) but 4 h after transfection, Huh7 cells were treated with Molidustat at 25 µM. After 48 h of culture, BNIP3, SERPINE1, LDHA and EPO expression was determined by RT-qPCR using RPL13A as housekeeping gene. Means ± SEM of four experiments. Data were normalized to cells transfected with control siRNA and stimulated with Molidustat. Means ± SEM of 4 experiments. *p < 0.05, **p < 0.01, ***p < 0.001; one sample t-test using 100% as reference and Holm-Šidák correction for multiple testing

To evaluate the role of HIF-1/2α in the transcriptional response to Molidustat, Huh7 cells were transfected with siRNA targeting HIF-1α or HIF-2α mRNA to suppress their expression. After Molidustat stimulation, BNIP3, SERPINE1, LDHA and EPO mRNA levels were quantified by RT-qPCR. The siRNAs suppressed the expression of HIF-1α or HIF-2α mRNA or both when co-transfected (Fig. 2A). HIF-1α knockdown prevented the induction of BNIP3 and LDHA mRNA by Molidustat, whereas SERPINE1 was unaffected and EPO was significantly enhanced (Fig. 2B). On the contrary, HIF-2α knockdown showed no effect on BNIP3 and LDHA, only partially impaired SERPINE1 expression, but totally suppressed the induction of EPO by Molidustat (Fig. 2B). Only the combination of HIF-1α and HIF-2α siRNAs abrogated the expression of these four genes in Molidustat-treated cells (Fig. 2B). Overall, this demonstrates that both HIF-1α and HIF-2α participate in the response of Huh7 cells to Molidustat.

### Molidustat upregulates enzymes of the glycolytic pathway in Huh7 cells

**Fig. 3:**
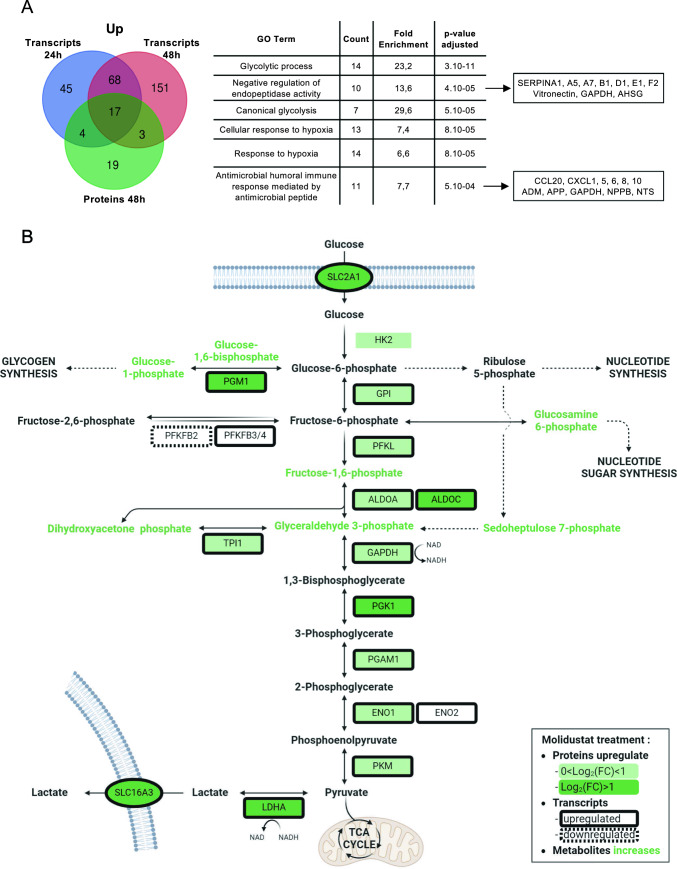
Molidustat upregulates the expression of glycolytic enzymes in Huh7 cells. **A** Venn diagram showing upregulated transcripts at either 24 h or 48 h of stimulation with Molidustat (Log_2_(FC) > 1; p < 0.05), and proteins upregulated by Molidustat after 48 h of culture (Log_2_(FC) > 1; p < 0.05). The right panel shows GO terms that are enriched in the gene set corresponding to the union of upregulated transcripts and proteins. **B** Glycolysis pathway showing proteins upregulated by Molidustat treatment (light green for 0 < Log_2_(FC) < 1 and dark green for Log_2_(FC) > 1; p < 0.05). Black frames indicate transcripts that are upregulated after 24 or 48 h of Molidustat treatment (Log_2_(FC) > 1 and p < 0.05). Black dashed frames indicate transcripts that are downregulated (Log_2_(FC) < − 1 and p < 0.05). Displayed enzyme were associated to GO “Glycolytic process” and “fructose metabolic process”. Glucose transporter SLC2A1 (GLUT1) and lactate transporter SLC16A3 (MCT4) which are upregulated at transcriptional and protein levels were also added on the figure. Metabolites whose concentration increased in Molidustat-treated Huh7 cells are highlighted in green (p < 0.05; See Supplementary Fig. 2 for details). Created with BioRender.com

Complementary to the transcriptomic profile described above, we analyzed by liquid chromatography-tandem mass spectrometry (LC–MS/MS) the proteome of Huh7 cells treated with Molidustat. This was performed after 48 h of stimulation to give cells sufficient time to translate upregulated transcripts and remodel their metabolic machinery. Compared to untreated cells, 43 proteins were found to be upregulated by Molidustat and 84 were downregulated (Tables S5 and S6, respectively). We first analyzed upregulated proteins and compared them to induced transcripts at 24 h and 48 h of treatment. As shown in Fig. 3A (left panel), 56% of the induced proteins also had their transcripts significantly upregulated by Molidustat, which is consistent with the induction of HIFs and their target genes. However, the partial overlap of transcriptomic and proteomic data also highlights the benefit of combining both approaches. Indeed, beyond the false-negative rate inherent to these techniques, not all upregulated transcripts are translated to proteins and conversely, proteins can be upregulated by post-translational regulation. The union of upregulated transcripts and proteins was then used to search the GO database for enriched functional annotations (Fig. 3A; right panel). Hypoxia-related terms were in the top list, and other functional annotations were also enriched including “Negative regulation of endopeptidase activity”. Of the 10 genes tagged with this term, 7 were SERPINs, i.e. extracellular serine protease inhibitors that regulate coagulation and fibrinolysis, and protect tissues from the proteases of activated immune cells. Genes associated to the “Antimicrobial humoral immune response mediated by antimicrobial peptide” were also overrepresented, including hypotensive factors (ADM, NPPB, NTS) and chemokines (CCL20, CXCL1, 5, 6, 8, 10). Altogether, this reflects the connections of the hypoxia pathway with the regulation of blood flow (coagulation, blood pressure) and the inflammatory response (chemokines). At the top of the list, the most enriched GO term was the glycolytic process with 14 genes associated to glycolysis induced by Molidustat, in line with previous reports showing that hypoxia induces genes encoding glycolytic enzymes [5].

As depicted in Fig. 3B, many enzymes involved in glycolysis were induced according to transcriptional data (black frames). Proteomic data confirmed this induction with most of the glycolytic enzymes being significantly upregulated at protein level (green boxes), with some of them being increased by at least twofold (SLC2A1, PGM1, ALDOC, PGK1) (dark green boxes). HK2, the first rate-limiting enzyme of glycolysis that phosphorylates glucose, was also induced at protein level (Log_2_(FC) = 0.81; p = 0.00026), although the upregulation of the transcripts was not statistically significant. Altogether, this shows that Molidustat stimulates the expression of enzymes involved in glycolysis. Moreover, the expression of both lactate synthesis enzyme (LDHA) and transporter (SLC16A3) were also strongly increased. The transcriptomic and proteomic profiling was complemented by untargeted metabolomic analysis of Molidustat-treated *vs* untreated cells (Supplementary Fig. 2A). Pathway enrichment analysis of the list of differentially expressed metabolites with MetaboAnalyst showed an association with glycolysis, gluconeogenesis, the pentose phosphate pathway and the Warburg effect (Supplementary Fig. 2B). More specifically, Molidustat increased intracellular levels of metabolites that are upstream of glyceraldehyde 3-phosphate dehydrogenase (GAPDH) in the glycolytic pathway (Supplementary Fig. 2A and Fig. 3B). Metabolites involved in glycogenesis, the pentose phosphate and the nucleotide sugar pathways were also increased, while intracellular levels of pyruvate or lactate were stable (Supplementary Fig. 2C). These results show an overall induction of glycolytic enzymes by Molidustat with differential effects on metabolites depending on whether they are either upstream or downstream of GAPDH, suggesting the presence of a bottleneck at this level.

### Remodeling of the mitochondrial network and decreased activity of the respiratory chain

**Fig. 4:**
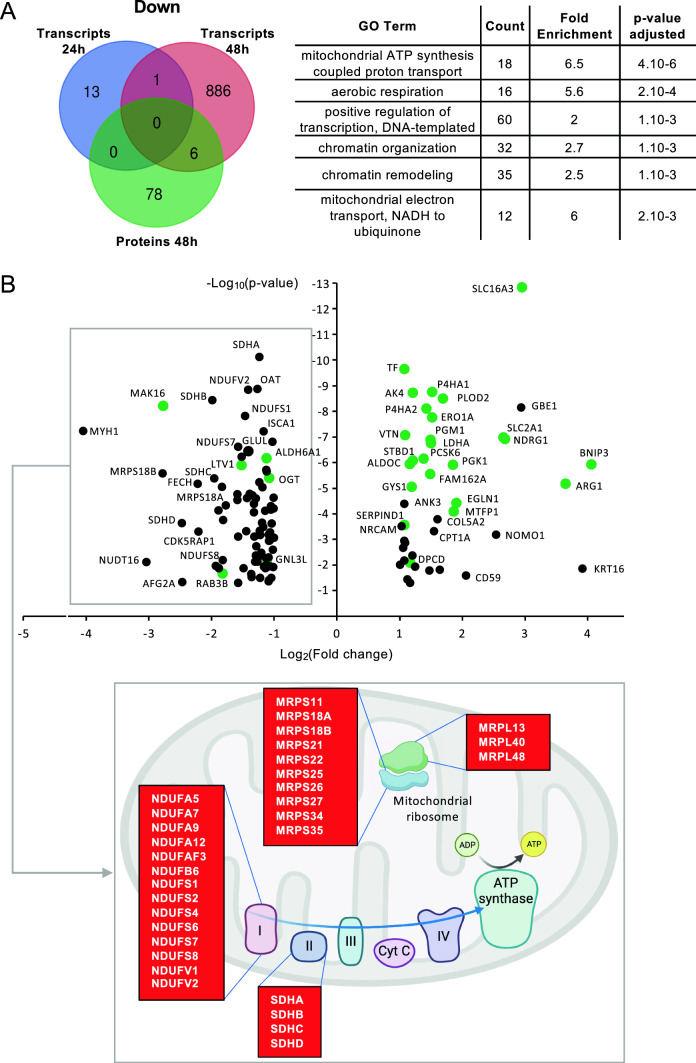
Molidustat treatment downregulates the expression of mitochondrial enzymes. **A** Venn diagram showing downregulated transcripts at either 24 h or 48 h of stimulation with Molidustat (Log_2_(FC) < − 1; p < 0.05), and proteins downregulated by Molidustat after 48 h of culture (Log_2_(FC) < − 1; p < 0.05). The right panel shows GO terms that are enriched in the gene set corresponding to the union of downregulated transcripts and proteins. **B** Dot plot showing proteins that are down or upregulated by Molidustat treatment (Log_2_(FC) < − 1 or > 1; p < 0.05). Matching transcripts that are also down or upregulated at either 24 or 48 h of treatment (Log_2_(FC) < − 1 or > 1; p < 0.05) are highlighted in green. The lower panel shows downregulated proteins of the mitochondrial ribosome and respiratory chain (Log_2_(FC) < − 1; p < 0.05). Created with BioRender.com

We then analyzed downregulated proteins and compared them to downregulated transcripts. In total, 984 factors were downregulated at transcriptional or protein level (Fig. 4A; left panel). Functional annotation analysis showed an enrichment for factors involved in chromatin remodeling, transcription and mitochondrial respiratory chain (Fig. 4A; right panel). Quite surprisingly, only 0.7% of the 906 transcripts whose expression was suppressed by Molidustat matched downregulated proteins (Fig. 4A; left panel). The fact that 10% of these transcripts are non-coding RNA contributes to this low overlap with protein expression. For the rest, proteins are probably stable enough to have their expression maintained, at least for 48 h, while the corresponding transcripts are down-regulated. Figure 4B (upper panel) shows this decorrelation with more details. Up and down regulated proteins appear in green when their corresponding transcripts are also up or down-regulated, respectively. While most up-regulated proteins match up-regulated transcripts, it is not the case for down-regulated proteins since 78 proteins are downregulated without a specific regulation at transcriptional level. These proteins are thus suppressed independently of transcriptional regulation, suggesting that Molidustat activates another mechanism leading to their degradation.

**Fig. 5:**
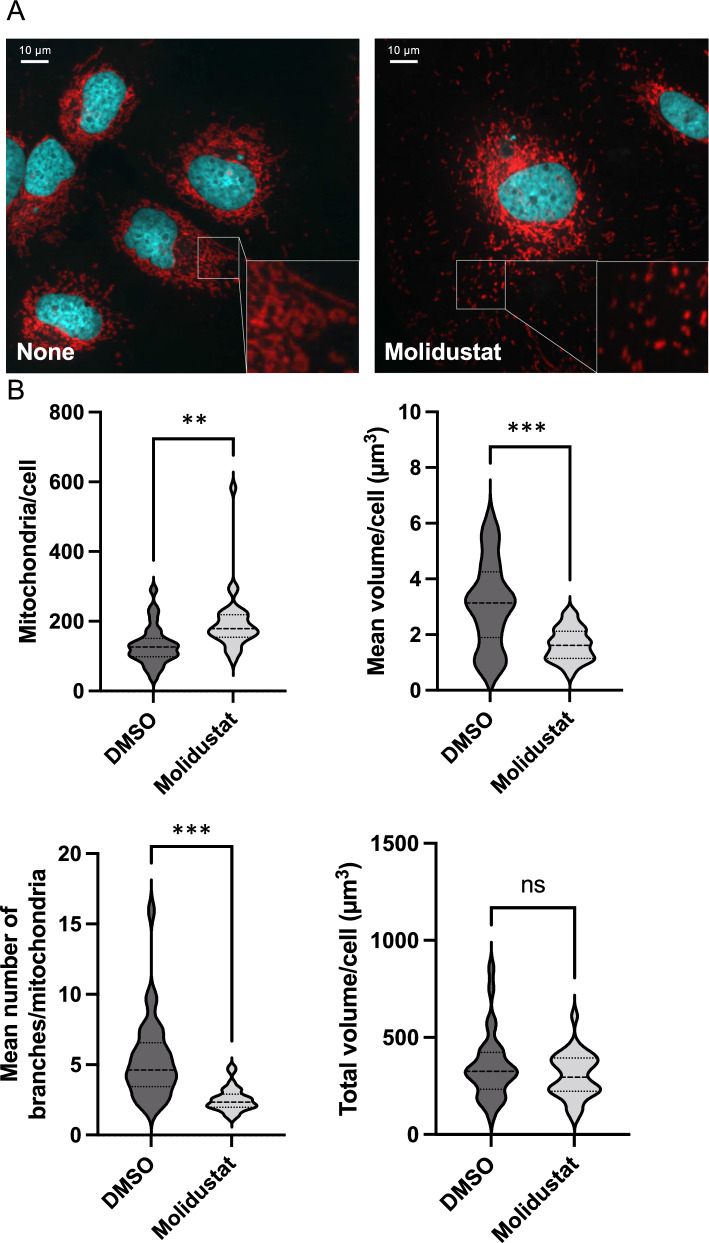
Mitochondrial network is remodeled by Molidustat treatment. **A** Huh7 cells were treated with Molidustat (25 µM) or DMSO alone for 48 h. Mitochondria (red) and nuclei (cyan) were stained with MitoTracker Red and Hoechst, respectively. Representative Z-stack projections of Huh7 cells obtained by fluorescence microscopy (× 40). **B** Analysis of the mitochondrial network. A total of 30 cells were analyzed to determine the number of mitochondria per cell, the mean volume of mitochondria per cell, the mean number of branches and the total volume of mitochondria per cell. **p < 0.01, ***p < 0.001; unpaired t-test

We thus analyzed downregulated transcripts and proteins separately for enriched functional annotations. Although downregulated transcripts were enriched for chromatin remodeling and transcriptional regulation, downregulated proteins were enriched for proteins involved in mitochondrial respiration and translation (Supplementary Fig. 3). Multiple components of the mitochondrial ribosome as well as subunits of Complex I and II of the respiratory chain were downregulated (Fig. 4B). When loosening our criteria for downregulated proteins (Log_2_(FC) < 0; p < 0.05), it appeared that several enzymes of the TCA cycle and all four complexes of the respiratory chain were also affected (Supplementary Fig. 4A). This was associated with the cleavage of VDAC (voltage-dependent anion channel) which was previously linked to cellular response to hypoxia and resistance to apoptosis (Supplementary Fig. 4B; [48]). These results show that Molidustat has a strong impact on the expression of mitochondrial components. The downregulation of mitochondrial proteins was associated to morphological changes as assessed by fluorescence microscopy (Fig. 5A). Indeed, the number of mitochondria per cell increased while the mean volume of mitochondria decreased (Fig. 5B). Besides, the mean number of branches per mitochondria decreased in Molidustat-treated cells (Fig. 5B). This demonstrates that the mitochondrial network was fragmented as previously described in HCC cells under hypoxia [49]. However, the total volume of mitochondria was unchanged (Fig. 5B). This led us to further investigate mitochondrial functions in Molidustat-treated cells.

**Fig. 6:**
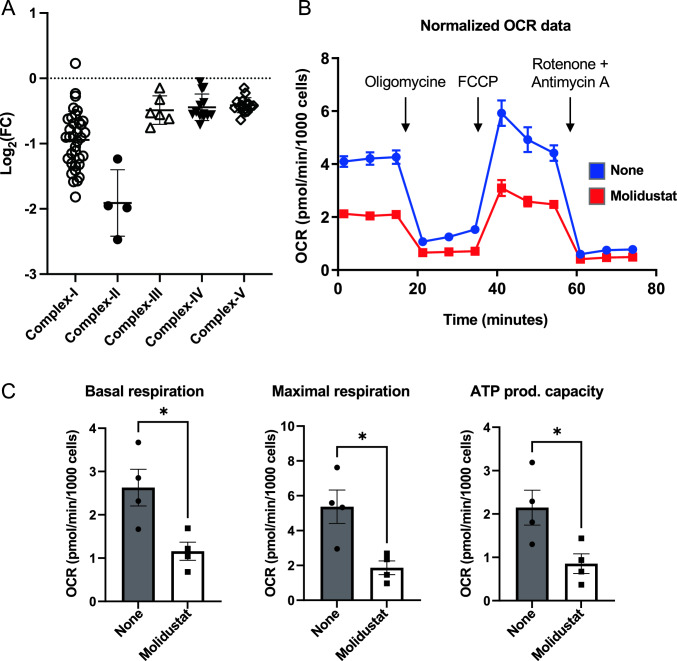
Molidustat inhibits mitochondrial respiration. **A** Effect of Molidustat on the expression of individual subunits of Complex I to IV of the respiratory chain as determined by proteomics analysis. Each dot corresponds to a different subunit of each complex. **B**, **C** Huh7 cells were treated for 24 h with Molidustat (25 µM) or DMSO alone (None). Oxygen consumption rate (OCR) were determined with a Seahorse analyzer. **B** Monitoring for one experiment. **C** Mitochondrial basal and maximal respiration, ATP production and basal acidification rate were determined from 4 independent experiments. Mean ± SEM; *p < 0.05; paired t-test

To visualize the effect of Molidustat on individual subunits of Complex I to IV of the respiratory chain, changes in their expression were determined from proteomics data and plotted on the same graph (Fig. 6A). Complexes I and II were more affected than Complexes III, IV and V. To evaluate directly the effect of Molidustat on mitochondrial respiration, we measured the oxygen consumption rate (OCR) of cells treated or not with Molidustat for 48 h (Fig. 6B and C). Basal and maximal respiration as well as the mitochondrial ATP production capacity were significantly decreased (Fig. 6C). Overall, this shows that in line with the downregulation of mitochondrial enzymes of the respiratory chain, mitochondrial respiration is reduced upon Molidustat treatment.

### Molidustat inhibits cellular proliferation without affecting viability

**Fig. 7:**
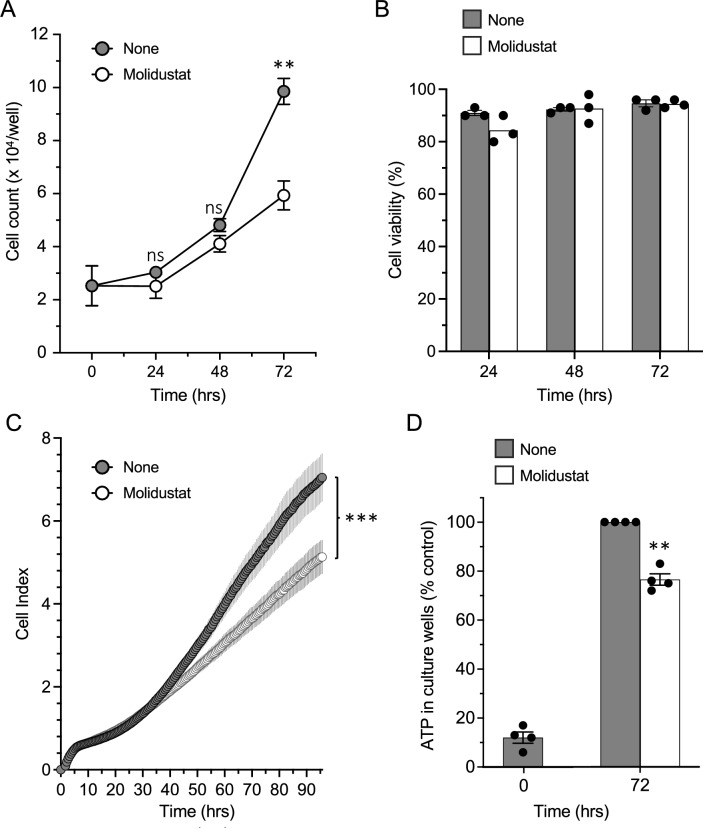
Molidustat inhibits the proliferation of Huh7 cells. **A**, **B** Huh7 cells were cultured with Molidustat (25 µM) or control DMSO (None) for 24, 48 or 72 h. The number of viable cells (**A**) and percentage of viability (**B**) were determined at indicated timepoints by automated counting and trypan blue exclusion. Means ± SEM of one experiment in triplicate. **p < 0.01; two-way ANOVA with post-hoc test and Šidák correction for multiple testing. **C** Huh7 cells were cultured with Molidustat (25 µM) or control DMSO (None) and cell proliferation was determined by impedance measurement every 15 min from t = 0 h to 96 h. Means ± SEM of six experiments in duplicate. Area under the curves (AUC ± SE) were calculated and compared by unpaired t-test. ***p < 0.001. (D) Huh7 cells were treated with Molidustat (25 µM) or control DMSO (None). ATP level in culture wells were determined at t = 0 h and after 72 h of culture. Data were normalized to untreated control (None). Means ± SEM of 4 experiments in triplicate. **p < 0.01; One sample t-test with 100% as reference

**Fig. 8:**
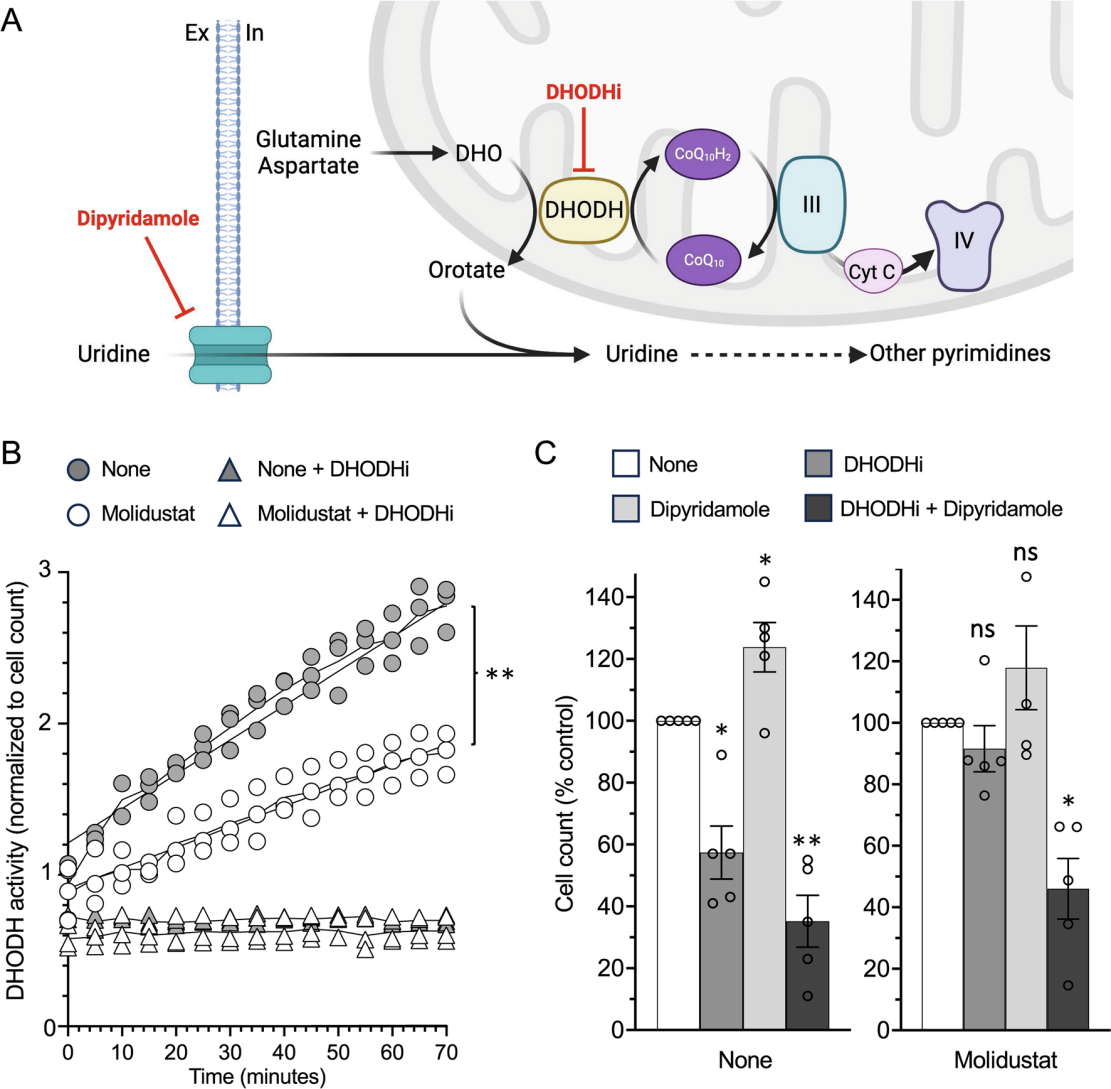
Molidustat-treated cells can use either uridine salvage or de novo biosynthesis to fulfill their needs in pyrimidines. **A** Schematic showing that uridine comes from extracellular import and de novo biosynthesis which involves DHODH, a mitochondrial enzyme coupled to the respiratory chain. Dipyridamole and DHODHi inhibit uridine uptake and the de novo biosynthesis pathway, respectively. Created with BioRender.com. **B** Huh7 cells were treated for 48 h with DMSO alone (None; grey circles) or Molidustat (25 µM; opened circles). Cells were harvested, and DHODH activity in corresponding protein lysates was determined. To control assay specificity, the DHODH inhibitor IPPA17-A04 was added to protein lysates at 2 µM. Means ± SEM of 3 experiments. ***p < 0.001; Simple linear regression with slope comparison. **C** Huh7 cells were treated with DMSO alone (None), DHODH inhibitor IPPA17-A04 (1 µM) or Dipyridamole (2 µM) in the absence or presence of Molidustat (25 µM). After 72 h, cell counts were determined by Hoechst staining and quantification of the fluorescence signal. Data were normalized to untreated control (None) without (left panel) or with Molidustat (right panel). Means ± SEM of 5 experiments in triplicate. *p < 0.05, **p < 0.01; One sample t-test using 100% as reference and Holm-Šidák correction for multiple testing

The metabolic reprogramming described above led us to analyze the consequences of Molidustat treatment on cell proliferation and viability. Huh7 cells were cultured for 24, 48 or 72 h with or without Molidustat. Cell growth and viability were then determined by cell counting and trypan blue exclusion. Molidustat inhibited cellular proliferation but did not induce cell death (Fig. 7A and B). The absence of cellular toxicity was confirmed by measuring changes in membrane integrity using CellTox Green, a cyanine dye that is excluded from living cells but stains the DNA of dead cells (Supplementary Fig. 5). Indeed, Molidustat did not increase the CellTox Green signal in Huh7 cells treated for 24, 48 or 72 h with this drug. To confirm the effect of Molidustat on proliferation, we monitored cell growth with the xCELLigence Real-Time Cell Analysis (RTCA) system. In this assay, cells are grown in microplates with gold electrodes embedded at the bottom of the wells to determine the density of adherent cells by impedance. Molidustat-treated cells still proliferate but slowly compared to untreated cells (Fig. 7C). Finally, ATP level in cells, which is used as an indicator of metabolic activity, decreased in Molidustat-treated cultures in proportion to the reduced proliferation observed by counting cells or by RTCA (Fig. 7D). Overall, this shows that Molidustat reduces cell proliferation but does not stop it. These results suggest that despite significant remodeling of the mitochondrial network, cellular respiration in Molidustat-treated cell remains sufficient to support metabolic functions essential for cell survival and proliferation. This notably includes the de novo biosynthesis of pyrimidines which is necessary for DNA replication when extracellular sources of pyrimidines are insufficient. Indeed, a key role of cellular respiration is to support the activity of dihydroorotate dehydrogenase (DHODH) which catalyzes the fourth rate-limiting step of the de novo pyrimidine biosynthesis pathway [1, 2]. This mitochondrial enzyme converts DHO to orotate by using ubiquinone (CoQ_10_) as cofactor (Fig. 8A). This produces ubiquinol (CoQ_10_H_2_), as do Complexes I and II of the respiratory chain and other mitochondrial dehydrogenases (GPD2, ETFHD, etc.; [50]). Complex III and the downstream part of the respiratory chain is thus required to re-oxidize CoQ_10_H_2_ back to CoQ_10_ and to maintain DHODH activity. We therefore analyzed the activity of DHODH in Molidustat-treated Huh7 cells.

### Molidustat-treated cells have increased flexibility in their uridine supply source

First, we compared the metabolome of cells treated with either Molidustat or pharmacological inhibitors of DHODH (DHODHi) like IPPA17-A04 [51] or BAY-2402234 [52]. In Huh7 cells, DHODHi treatment led to a major decrease in pyrimidine levels (UDP, CTP, CDP, uridine) and increased concentration of precursors such as carbamoyl-aspartate and DHO (Supplementary Fig. 6). This was not the case in Molidustat-treated Huh7 cells in which, of all pyrimidines, only 2′-deoxycytidine level decreased. Besides, DHO did not accumulate but decreased in these cells (Supplementary Fig. 2). Altogether, this suggests that DHODH is still functional in these cells. To explore more directly DHODH activity in Molidustat-treated Huh7 cells, cellular lysates containing mitochondria were incubated with DHO and decylubiquinone as substrate and cofactor, respectively [40]. Orotate synthesis was then measured every 5 min for 70 min. As shown in Fig. 8B, DHODH activity was significantly reduced in lysates of Molidustat-treated cells compared to untreated cells but maintained at an intermediate level, while DHODH activity was completely abolished by the addition of a DHODHi (IPPA17-A04). This shows that upon Molidustat treatment, DHODH enzyme was still able to catalyze the fourth rate-limiting step of de novo pyrimidine biosynthesis.

We then compared the dependence of Huh7 cells on this metabolic pathway for their proliferation when treated or not with Molidustat. Huh7 cells were grown for 72 h with or without Molidustat in the presence of IPPA17-A04 as DHODHi and/or dipyridamole (DPD), a nucleoside transport inhibitor preventing uridine uptake from the extracellular compartment for the pyrimidine salvage pathway (Fig. 8A). In the absence of Molidustat, DHODH inhibition reduced cell count by 40%, whereas DPD alone had no inhibitory effect (Fig. 8C; left panel). When DHODHi and DPD were combined, the cell count was further reduced by 65% compared to untreated cells. Overall, this shows that the de novo pyrimidine biosynthesis pathway contributes to the proliferation of Huh7 cells, with a maximal inhibitory effect observed only when DHODHi and DPD are combined to block all sources of uridine. In the presence of Molidustat, cellular proliferation was reduced as described in Fig. 7. However, neither DHODHi nor DPD addition was able to further reduce the proliferation of Molidustat-treated cells (Fig. 8C; right panel). Only the combination of these two inhibitors inhibited the proliferation of Molidustat-treated cells. Therefore, in the presence of Molidustat, Huh7 cells fully satisfy their needs in pyrimidines by using either the de novo synthesis pathway or uridine uptake. Finally, the addition of uridine at high concentrations in the culture medium did not revert the inhibitory effect of Molidustat on cell growth, confirming that the limited proliferation of Molidustat treated cells is not due to a lack of pyrimidines (data not shown). Overall, this shows that DHODH is still active in Huh7 cells treated with Molidustat. It also demonstrates that at the expense of slower proliferation, Molidustat-treated cells have greater flexibility in their uridine supply source.

### Molidustat activates pyruvate metabolism to overcome Complex III inhibition by antimycin

**Fig. 9:**
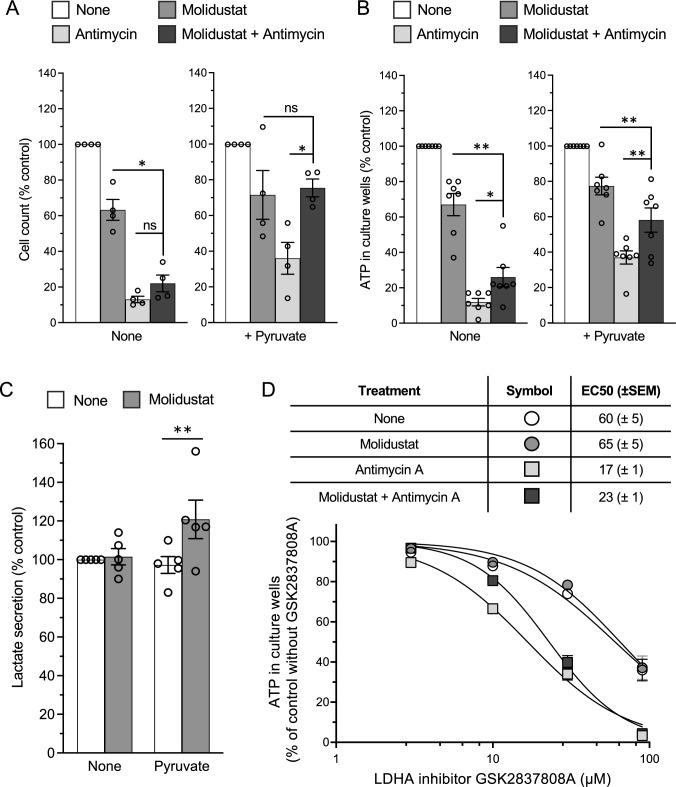
Proliferation of Huh7 cells is inhibited by Complex III inhibitor antimycin, but is restored in the presence of pyruvate and Molidustat. **A**, **B** Huh7 cells were treated with DMSO alone, Molidustat (25 µM) and antimycin (1 µM) in the absence or presence of pyruvate (1 mM). After 72 h, cell counts were determined by Hoechst staining and quantification of the fluorescence signal (**A**) and ATP level in culture wells that reflects the number of viable cells was measured (**B**). Data were normalized to untreated control (None) without (left panels) or with pyruvate (right panels). Means ± SEM of four (**A**) and seven (**B**) experiments in triplicate. *p < 0.05, **p < 0.01; paired t-test. **C** Huh7 cells were treated for 72 h with Molidustat (25 µM) or DMSO alone (None), with or without 1 mM Pyruvate. Lactate accumulation in the extracellular medium was quantified and reported to control (None). Means ± SEM of five experiments in triplicate. **p < 0.01; paired t-test. **D** Huh7 cells were grown in culture medium supplemented with pyruvate (1 mM), LDHA inhibitor GSK2837808A at 3, 10, 30 and 90 µM, and DMSO (None), Molidustat (25 µM), antimycin (1 µM) or Molidustat plus antimycin. After 72 h, ATP level in culture wells was determined. For DMSO, Molidustat, antimycin or Molidustat plus antimycin, data were normalized to the control condition without GSK2837808A. Best fit curves as well as EC_50_ values were calculated from the dose–response of GSK2837808A. Data correspond to the mean of six experiments in triplicate

In addition to ATP synthesis by oxidative phosphorylation (OXPHOS) and the re-oxidation of CoQ_10_H_2_ back to CoQ_10_, mitochondrial respiration is also essential to maintain the NAD^+^ /NADH ratio which is essential for the metabolic activity of many pathways. In particular, NAD^+^ is used by the malate dehydrogenase (MDH1)/aspartate aminotransferase (GOT1) pathway to produce aspartate which is required for the biosynthesis of purines and pyrimidines and for maintaining cellular proliferation [3, 4]. To test the dependence of Molidustat-treated cells on mitochondrial respiration for their proliferation, cells were cultured for 72 h with combinations of Molidustat and/or Complex III inhibitor antimycin. The number of cells was determined by nuclei staining with Hoechst before measuring fluorescence signal (Fig. 9A; left panel) while their metabolic activity was monitored by measuring ATP level (Fig. 9B; left panel). Antimycin drastically decreased the number of cells and ATP level, providing evidence that cellular proliferation and/or viability were strongly impaired. Interestingly, Molidustat slightly attenuated the effect of antimycin on ATP level and cell numbers although without reaching statistical significance, suggesting some benefit on cell survival and/or proliferation (Fig. 9A, B; left panels). Nevertheless, Complex III activity is essential for the proliferation of Huh7 cells treated or not with Molidustat. This demonstrates that despite the induction of the hypoxia response pathway, the residual mitochondrial respiration detected in these cells is paramount for maintaining cell proliferation.

The conversion of pyruvate to lactate by LDHA is another way to re-oxidize NADH to NAD^+^ when mitochondrial respiration is inhibited. However, the net production of pyruvate by glycolysis is only sufficient to regenerate the NAD^+^ used by GAPDH for the maintenance of glycolysis itself (Fig. 3B). Interestingly, it has been demonstrated in several cancer cell lines that pyruvate supplementation can be used to maintain the NAD^+^ /NADH ratio, and to restore proliferation when Complex III of the respiratory chain is inhibited [3, 4]. Since Molidustat upregulates LDHA (Fig. 3B), we determined whether pyruvate conversion to lactate is improved as well as its ability to restore cellular proliferation when Complex III is inhibited. Consistently with metabolomic data (Fig. 3B and Supplementary Fig. 2), lactate secretion was unchanged by Molidustat treatment in a culture medium without pyruvate (Fig. 9C). Similarly, pyruvate supplementation alone had no effect. However, when pyruvate was combined with Molidustat treatment, lactate secretion increased. These results indicate that lactate secretion is limited in Molidustat-treated Huh7 cells by the availability of pyruvate.

We thus determined whether the addition of pyruvate to culture medium was able to revert the effect of Complex III inhibition on cell proliferation, and analyzed the impact of Molidustat treatment. The addition of pyruvate to culture medium attenuated the effect of Complex III inhibition by antimycin, although the number of cells and ATP level remained lower compared to control (Fig. 9A, B; right panels). Interestingly, the combination of pyruvate and Molidustat strongly attenuated, or even abolished, the effect of antimycin when considering ATP level or cell count, respectively. We wanted to extend this observation to another PHDi and used Roxadustat which was validated for the induction of HIF1 and HIF2 in Huh7 cells. First, we verified that Roxadustat does not induce cell death using the CellTox Green assay (Supplementary Fig. 5). Secondly, we found that Roxadustat, like Molidustat, was also able to counteract the effect of antimycin (Supplementary Fig. 7). Overall, this shows that PHDi increase the ability of Huh7 cells to use extracellular pyruvate to overcome Complex III inhibition and to restore cellular proliferation.

To strengthen this conclusion, we assessed the dependence of Huh7 cells on LDHA when cultured in the presence of antimycin. Huh7 cells were grown in a culture medium supplemented with pyruvate and increasing concentrations of the LDHA inhibitor GSK2837808A. Their metabolic activity was determined by measuring ATP levels and results were normalized to the untreated control to calculate the EC_50_ of GSK2837808A in the different culture conditions (Fig. 9D). EC_50_ was estimated to 60 μM in untreated cultures, and was not affected by the addition of Molidustat to culture medium (EC_50_ = 65 μM). In contrast, the presence of antimycin greatly increased cellular dependence on LDHA as assessed by the lower EC_50_ which was estimated to 17 μM. Interestingly, similar results were obtained when combining antimycin treatment with Molidustat (EC_50_ = 23 μM). This shows that once Complex III is inhibited with antimycin, dependence on LDHA increases. Furthermore, although Molidustat improves cell proliferation and metabolic activity in antimycin-treated cultures (Fig. 9A, B), dependence on LDHA remains high.

**Fig. 10:**
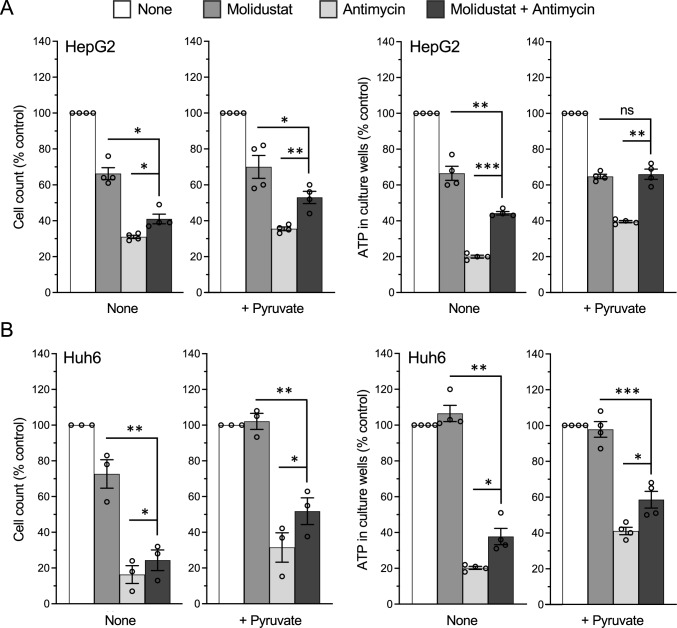
Molidustat counteracts the effects of Complex III inhibition in HepG2 and Huh6 cells. **A** HepG2 cells were treated with DMSO alone, Molidustat (25 µM) and antimycin (1 µM) in the absence or presence of pyruvate (1 mM). After 72 h, cell counts were determined by Hoechst staining and quantification of the fluorescence signal (left panels) and ATP level in culture wells (right panels). Data were normalized to untreated control (None) without (left panels) or with pyruvate (right panels). Means ± SEM of four experiments in triplicate. **B** Same experiment as in (**A**) performed on Huh6 cells. Means ± SEM of three (Cell count) or four (ATP level) experiments in triplicate. *p < 0.05, **p < 0.01, ***p < 0.001; paired t-test

Finally, we evaluated in other liver cancer cell lines the ability of Molidustat to counteract the effects of Complex III inhibition. We thus repeated the same experiments as in Fig. 9A, B on hepatoblastoma-derived cell lines HepG2 and Huh6 (Fig. 10A, B, respectively). We first verified that Molidustat induced the accumulation of HIF-1α and HIF-2α proteins in these two cell lines (Supplementary Fig. 8A and B). We then determined the effect of Molidustat on these cells cultured for 72 h in the absence or presence of antimycin to block Complex III. For both cell lines, the number of cells and ATP levels were reduced by antimycin, and the addition of Molidustat partially counteracted this inhibitory effect (Fig. 10A, B). The presence of pyruvate further improved this effect of Molidustat. Thus, HepG2 and Huh6 cells behaved essentially in the same way as Huh7 cells since Molidustat confers them resistance to antimycin, especially when pyruvate was added to culture medium.

## Discussion

**Fig. 11:**
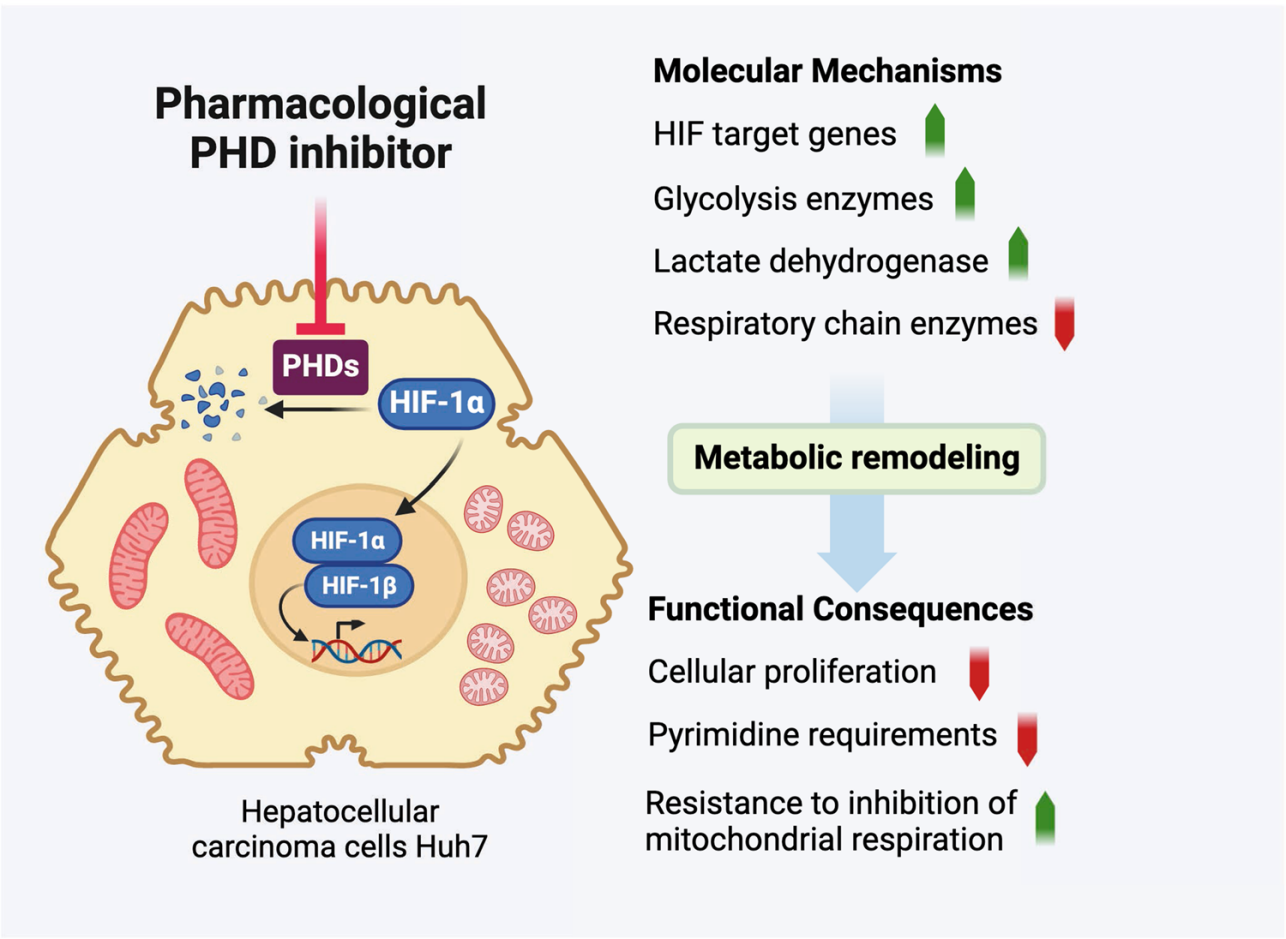
Visual summary of the metabolic response of Huh7 cells to Molidustat. Created with BioRender.com

PHDi that stabilize HIF-1α expression and activate the hypoxia response pathway have been developed in the last decade for treating anemia, and several of these molecules are now in clinic. However, HIF-1 expression has been associated to poor prognosis for several types of cancer including HCC raising some concerns about the safety of these molecules. We were thus interested in studying the effect of two PHDi, Molidustat and Roxadustat, on Huh7 cells, which is one of the best characterized in vitro models of HCC. By using a multi-omics approach, we found that PHDi induce a profound remodeling of Huh7 metabolism (Fig. 11).

We first established that Molidustat and Roxadustat induces the hypoxia response as evidenced by the induction of HIF-1α and HIF-2α, and by the expression profile of reference hypoxia-response genes. Interestingly, we showed that cellular response to Molidustat depends both on HIF-1α and HIF-2α, in line with previous reports in other cell types showing that these two factors control distinct although partially overlapping sets of genes [53]. One key function of PHDi is to stimulate the expression of EPO that is altered in chronic kidney disease. After kidneys, the liver is a secondary source of EPO, and our results show that Huh7 cells have indeed the ability to produce EPO upon Molidustat treatment [54]. Genes encoding direct regulators of HIF-1/2α protein stability or activity were also induced as part of negative or positive feedback regulatory loops, including the HIF-prolyl hydroxylase EGLN1 (a.k.a PHD2), the E3 ubiquitin ligase WSB1 which targets VHL for proteasomal degradation [55] and CITED2 that competes with HIF-mediated transcription by interacting with the transcription coactivator complex p300-CBP [56]. Molidustat treatment was also associated to the induction of BNIP3 and BNIP3L, which are key HIF-induced genes involved in the fission of mitochondria and mitophagy [57–59], and MXI1 that inhibits mitochondrial biogenesis by repressing c-Myc activity [60]. Their induction well correlates with the remodeling of the mitochondrial network, the loss of mitochondrial proteins identified by proteomic analysis, and decreased cellular respiration (Figs. 5, 6). We also found that Molidustat induces inhibitors of cellular proliferation such as ERRFI1/MIG-6 [61] and CCNG2 [62] that could contribute to the slower proliferation of treated cells (Fig. 7). Finally, a large fraction of hypoxia-response genes which are induced by Molidustat correspond to enzymes related to glycolysis (Fig. 3). The hypoxia response pathway enables cellular adaption to low O_2_ pressure. In primary cells, glycolysis and lactagenesis is upregulated to maintain ATP synthesis when O_2_ is missing. Unless the culture medium was supplemented with pyruvate, lactate secretion was not affected by Molidustat treatment (Fig. 9C), suggesting that glycolysis was in fact not increased. This may be explained by the bottleneck observed in the glycolytic pathway at the GAPDH step (Fig. 3B). This critical step of glycolysis is dependent on the availability of the NAD^+^ cofactor which is recycled from NADH, especially by mitochondrial respiration. As the latter is inhibited although not suppressed by Molidustat (see below), it is likely that NAD^+^ becomes a limiting factor for several metabolic pathways including glycolysis. NAD^+^ can also be replenished by increasing lactate synthesis from pyruvate (Fig. 3) [63], however our data clearly show that pyruvate is also a limiting factor in Molidustat-treated cells.

Although Molidustat clearly stimulates a cluster of HIF-response genes, it is surprising that key factors that are well described for their role in cellular response to hypoxia were not induced, including several regulators of mitochondrial biogenesis and activity, and components of the serine biosynthesis pathway [11]. In HCC cells, HIF-1α induction by hypoxia has been reported to induce the transcriptional repressor HEY1, which in turn inhibits PINK1, a kinase that regulates mitochondria biogenesis and mitophagy [64]. Although HEY1 and PINK1 were modulated by hypoxia in Huh7 [64], Molidustat showed no effect on these genes in our experiments. Other hypoxia-inducible factors regulating mitochondrial activity are NDUF4AL2 and COX4-2 which inhibit Complex I and IV of the respiratory chain, respectively [65–67]. Again, they were not induced by Molidustat in Huh7 cells, nor was UQCC3, a Complex III subunit that participates to metabolic adaptation to hypoxia [68], and enzymes of the serine biosynthesis pathway (PHGDH, PSAT1, PSPH, SHMT2, MTHFD2, MTHFD1L; [69]). Overall, this suggests that induction of these genes requires hypoxia conditions that are not mimicked by pharmacological induction of the HIF pathway with PHDi. On the other hand, more than three quarters of Molidustat-induced genes were not assigned to hypoxia response either in the MSigDB or Gene Ontology databases. This is partly because these functional annotation databases are not necessarily exhaustive, especially when it comes to a specific tissue such as the liver. For example, SLC16A3, which plays a key role in the export of lactate, is well known for being induced by hypoxia but is not flagged as such in the two functional annotation databases we used [70]. The same applies to MTFP1, a key player in mitochondria dynamics, which is induced by hypoxia in neuroblastoma cells and contains putative HIF-response elements in its promoter [71]. Furthermore, many Molidustat-induced genes may not be tagged as hypoxia response gene simply because they are induced indirectly as a result of Huh7 metabolic reprogramming. Among these factors, we were particularly interested by ARG1 whose induction was detected both at transcriptional and protein levels. ARG1 cleaves arginine to produce urea and ornithine in the last step of the urea cycle. It is also a potent immunosuppressive enzyme which prevents the activation of antitumor lymphocytes, and hypoxia was previously reported to induce ARG1 expression through mir-210 induction in myeloid-derived suppressor cells [72]. However, and quite paradoxically, it has been shown that ARG1 is decreased in HCC tumors whereas arginine level is increased and plays a major role in tumor formation [73]. It would be interesting to determine whether Molidustat is able to induce ARG1 expression in HCC tumors in vivo. This could inhibit tumor growth by degrading arginine and promote the establishment of an immunotolerant microenvironment, with overall consequences difficult to predict on tumor development.

Another key aspect of the cellular response to Molidustat is the inhibition of mitochondrial respiration, which is expected upon induction of the hypoxia response pathway [11, 74]. We found that Molidustat induces pyruvate dehydrogenase kinases PDK1 and PDK3, whose function is to phosphorylate and inhibit pyruvate dehydrogenase (PDH), blocking pyruvate conversion to Acetyl-CoA which is fueling the TCA cycle [75–78]. Besides, Molidustat treatment is associated with a loss of mitochondrial proteins, especially ribosomal subunits and components of the respiratory chain (Figs. 4, 6A). Expression of the corresponding genes was not suppressed by Molidustat as shown by transcriptomic analysis (Fig. 4), consistent with the idea that these proteins could be degraded by mitophagy. Interestingly, Complex I and II subunits were more affected (Fig. 6A), suggesting the existence of selective degradation mechanisms of these complexes. Upon Molidustat treatment, mitochondrial network was also fragmented (Fig. 5), which is a well-known consequence of hypoxia [74]. Aside BNIP3 upregulation, this phenotype correlates with the transcriptional induction of MTFP1 and FUNDC2 that promotes mitochondrial fission in HCC [79, 80], whereas MFN1 that allows fusion [80] is suppressed. However, and despite this profound remodeling, mitochondria maintain some metabolic functions in Molidustat-treated cells, and we have shown especially that Complex III remains functional and essential for cell proliferation (Fig. 9). The de novo pyrimidine biosynthesis pathway, which also requires mitochondrial respiration to support DHODH activity, is also maintained at an intermediate level (Fig. 8). Although reduced, mitochondrial activity is essential to support the proliferation of Molidustat-treated cells as assessed by the cytostatic effect of antimycin or inhibitors of pyrimidine biosynthesis. Finally, we provide evidence that Molidustat increases the metabolic plasticity of Huh7 cells as a consequence of this global reprogramming. First, we found that the cellular demand for pyrimidines can be met either by uridine uptake or de novo synthesis, and this phenotype is probably due to slower proliferation of Molidustat-treated cells. Secondly, these cells have a greater capacity to metabolize exogenous pyruvate into lactate, probably due to the increased expression of LDHA. As a result, the addition of pyruvate to culture medium is able to restore cellular proliferation when the respiratory chain is inhibited with antimycin (Fig. 9). Lactagenesis could compensate for the loss of Complex I activity by maintaining the NAD^+^ /NADH ratio, which is essential for multiple metabolic reactions, in particular aspartate synthesis [2–4, 81]. Inhibition of Complex III with antimycin probably also leads to inhibition of DHODH activity and de novo pyrimidine biosynthesis, but as demonstrated (Fig. 8), extracellular uridine satisfies the requirement in pyrimidines of Molidustat-treated cells. Overall, this suggests that Molidustat could thus facilitate cell adaptation to lower O_2_ levels in the microenvironment as is notably the case in solid tumors. This holds potential relevance in vivo, considering that blood contains high concentrations of pyruvate (70–200 µM) and uridine (3–8 µM), which can be compared to 1 mM and ~ 0.5 µM in our experimental settings, respectively [82, 83].

Whether cellular reprogramming and metabolic changes induced by Molidustat and other PHDi could increase the risk of developing HCC is an open question. Interestingly, out of the 288 genes whose expression is induced by Molidustat, 51 are associated to poor prognosis of liver cancer (Protein Atlas database v23; [84]), which is significantly more than expected by chance when considering the whole human genome (17.7% vs 13%; p = 0.0217; Fisher’s exact test). These genes are involved in glycolysis (ENO1/2, PGK1, PKM, TPI1, GAPDH, ALDOA), immunity (CCL20, CXCL1/5/8), erythropoiesis (EPO), angiogenesis (VEGFA) and remodeling of the extracellular matrix (PLOD2). Conversely, only 4.8% of Molidustat-induced genes were associated to good prognosis of liver cancer. Other Molidustat-induced genes that are not directly associated to liver cancer in the Protein Atlas database also deserve to be mentioned. For example, SERPINE1 was previously reported to promote angiogenesis in HCC [85], and CXCL8 expression is associated to inflammation, metastasis and HCC progression [86]. Thus, Molidustat tends to promote the expression of genes associated to HCC development.

In conclusion, our results demonstrate that PHDi induce a profound metabolic remodeling in Huh7 cells and the expression of multiple genes associated to HCC development. It has been established that PHDi are beneficial to patients with chronic kidney diseases and other potential indications for this new class of drugs is under evaluation. Because of the theoretical risk associated to the chronic induction of HIFs, follow up studies have been implemented to monitor cancer-related adverse event in patients treated with these drugs [20]. Our data support careful monitoring of patients treated with PHDi, especially when they are at risk of HCC.

### Supplementary Information

Below is the link to the electronic supplementary material.

Supplementary file1 (PDF 1642 KB)

Supplementary file2 (XLSX 980 KB)

## Data Availability

The proteomics datasets generated during the current study are available in the PRIDE partner repository, with the dataset identifier PXD049964**.** Raw transcriptomic data are available on the Gene Expression Omnibus database (Accession number GSE242340). Other datasets are available from the corresponding author upon reasonable request.
