# Supplementary material for: Pharmacological induction of the hypoxia response pathway in Huh7 hepatoma cells limits proliferation but increases resilience under metabolic stress
Source: Cell Mol Life Sci. 2024 Jul 30;81(1):320. doi: 10.1007/s00018-024-05361-6 (PMC11335246; doi:10.1007/s00018-024-05361-6)
Supplement: Supplementary file 1 — Supplementary file1 (PDF 1642 KB) [file 18_2024_5361_MOESM1_ESM.pdf]

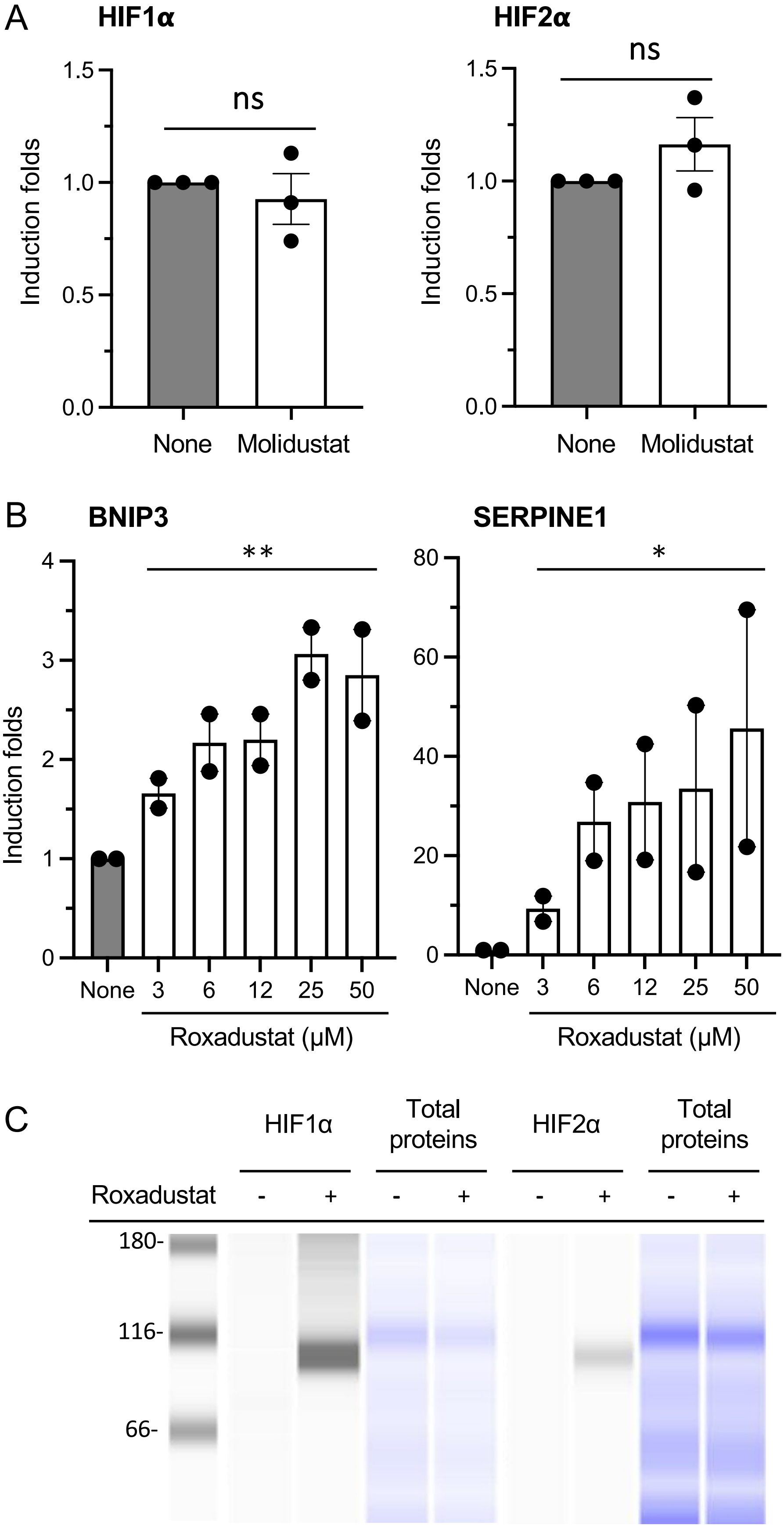

Supplementary Fig. 1 (A) Huh7 were stimulated for 48 h with Molidustat (25 μM) or DMSO alone (None). HIF-1α and HIF-2α expression was determined by RT-qPCR using RPL13A as housekeeping gene. Means ± SEM of three experiments. paired t-test. (B) Huh7 were stimulated for 48 h with Roxadustat at 3, 6, 12.5, 25 or 50 μM or DMSO alone (None). BNIP3 and SERPINE1 expression was determined by RT-qPCR using RPL13A as housekeeping gene. Means ± SEM of two experiments. *p<0.01, **p<0.01; Friedman test. (C) Huh7 cells were treated for 24 h with Roxadustat (25 μM) or DMSO alone, and HIF-1α and HIF-2α expression were determined by Jess analysis. Total proteins loaded in the capillaries are presented on the right panels.

A

Name	Log2FC	p-value
5-Aminolevulinic acid	-1,47	0,012
Dihydroorotic acid	-0,89	0,049
2′-Deoxycytidine	-0,78	0,011
3-Ureidopropionic acid	-0,67	0,048
Serine phosphate	-0,64	0,001
Malic acid	-0,60	0,024
Propionylcarnitine	-0,55	0,001
Acetylcarnitine	-0,52	0,005
Xanthosine	0,53	0,021
Glucose-1-phosphate	0,57	0,049
Sedoheptulose 7-phosphate	1,08	0,025
Glucosamine-6-phosphate	1,15	0,000
Fructose 1,6-bisphosphate	1,25	0,035
Glucose-1,6-bisphosphate	1,25	0,001
Glyceraldehyde 3-phosphate/ Dihydroxyacetone-phosphate	1,42	0,005
Creatine phosphate	1,93	0,010

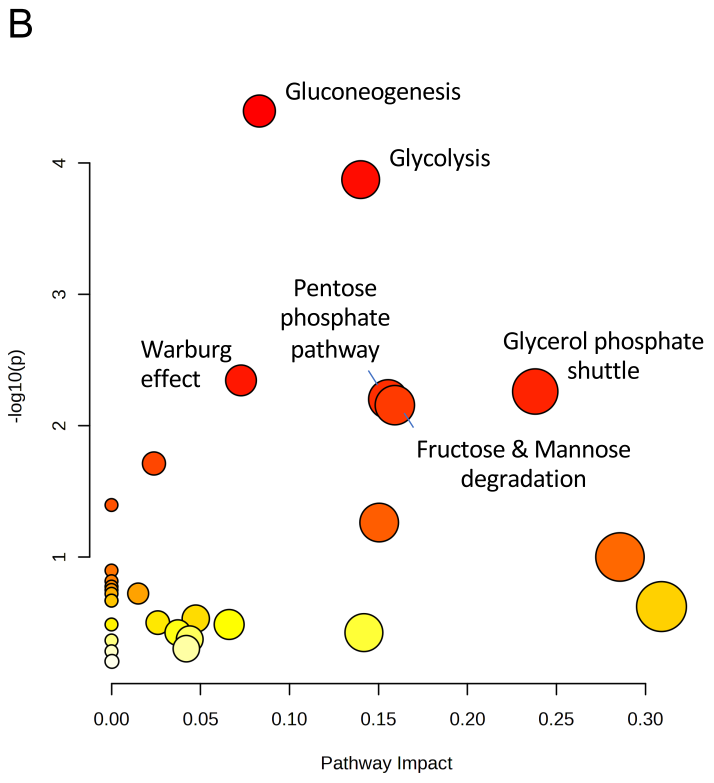

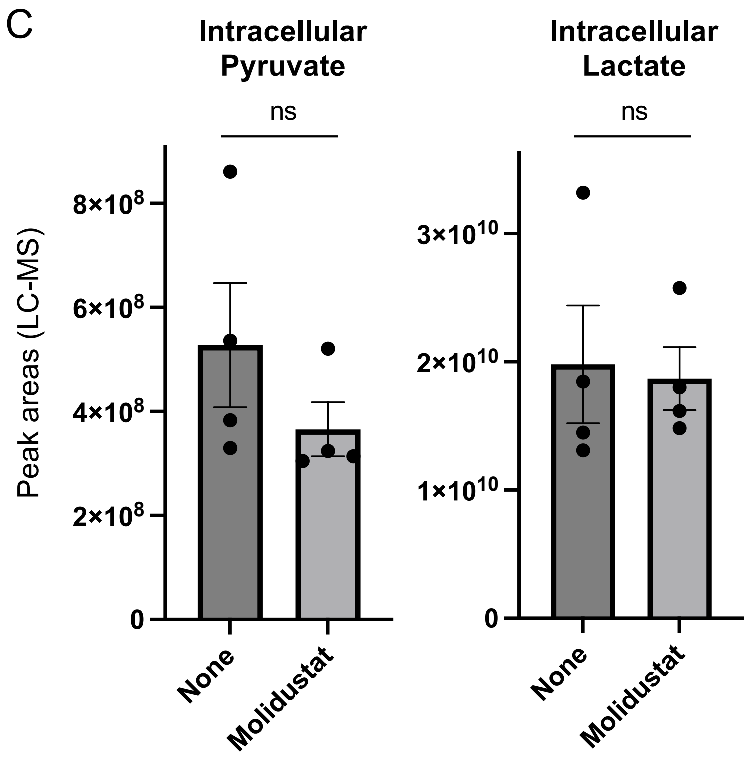

Supplementary Fig. 2 Metabolites that differ in their expression level in Molidustat-treated Huh7 cells compared to control cells. (A) Table showing metabolites that have differential relative levels quantified by LC-MS in Huh7 cells after 24 h of Molidustat treatment compared to control ($p<0.05$; Log2(FC)>0.5). P-values are calculated with a paired t-test. Decreased metabolites are colored in red and increased metabolites in green. (B) Pathway enrichment analysis performed on the list of differentially expressed metabolites from (A) with MetaboAnalyst 6.0 (https://www.metaboanalyst.ca/MetaboAnalyst/home.xhtml); Homo sapiens SMPDB pathway library; [84]). This module integrates enrichment analysis and pathway topology analysis. P-value is calculated from the enrichment analysis; pathway impact value is calculated from pathway topology analysis (node importance measure for topological analysis was based on relative betweenness centrality). (C) Histogram plot showing pyruvate and lactate levels quantified by LC-MS.

Supplementary Fig. 3 Enriched functional annotation in the downregulated transcript and protein sets. The functional enrichment analysis was performed with DAVID using the "Biological Process" (BP) annotation of the GO database.

Enriched functional annotations in the downregulated transcript set (Log2(FC)<-1; p-adj<0.05)

Sublist	Category	Term	RT	Genes	Count	%	P-Value	Benjamini
	GOTERM_BP_DIRECT	positive regulation of transcription, DNA-templated	RT		60	7,3	4,6E-8	1,6E-4
	GOTERM_BP_DIRECT	chromatin remodeling	RT		34	4,1	6,7E-7	1,1E-3
	GOTERM_BP_DIRECT	chromatin organization	RT		30	3,6	1,8E-6	2,1E-3
	GOTERM_BP_DIRECT	cellular response to DNA damage stimulus	RT		30	3,6	3,2E-6	2,7E-3
	GOTERM_BP_DIRECT	mRNA transport	RT		15	1,8	4,8E-6	2,8E-3
	GOTERM_BP_DIRECT	regulation of cell cycle	RT		28	3,4	5,0E-6	2,8E-3
	GOTERM_BP_DIRECT	cell division	RT		34	4,1	1,6E-5	7,9E-3
	GOTERM_BP_DIRECT	negative regulation of transcription, DNA-templated	RT		45	5,5	2,2E-5	9,2E-3
	GOTERM_BP_DIRECT	negative regulation of transcription from RNA polymerase II promoter	RT		63	7,7	1,0E-4	3,9E-2

Enriched functional annotations in the downregulated protein set (Log2(FC)<-1; p<0.05)

Sublist	Category	Term	RT	Genes	Count	%	P-Value	Benjamini
	GOTERM_BP_DIRECT	mitochondrial ATP synthesis coupled proton transport	RT		17	20,2	9,5E-25	3,6E-22
	GOTERM_BP_DIRECT	aerobic respiration	RT		15	17,9	1,5E-20	2,8E-18
	GOTERM_BP_DIRECT	mitochondrial translation	RT		15	17,9	2,7E-18	3,3E-16
	GOTERM_BP_DIRECT	mitochondrial electron transport, NADH to ubiquinone	RT		12	14,3	5,0E-17	4,7E-15
	GOTERM_BP_DIRECT	mitochondrial respiratory chain complex I assembly	RT		10	11,9	5,5E-12	4,1E-10
	GOTERM_BP_DIRECT	mitochondrial electron transport, succinate to ubiquinone	RT		4	4,8	7,0E-7	4,3E-5
	GOTERM_BP_DIRECT	mitochondrial ATP synthesis coupled electron transport	RT		4	4,8	8,2E-6	4,4E-4
	GOTERM_BP_DIRECT	respiratory electron transport chain	RT		4	4,8	1,0E-4	4,7E-3
	GOTERM_BP_DIRECT	tricarboxylic acid cycle	RT		4	4,8	4,2E-4	1,7E-2
	GOTERM_BP_DIRECT	ribosome biogenesis	RT		4	4,8	6,7E-4	2,5E-2

A

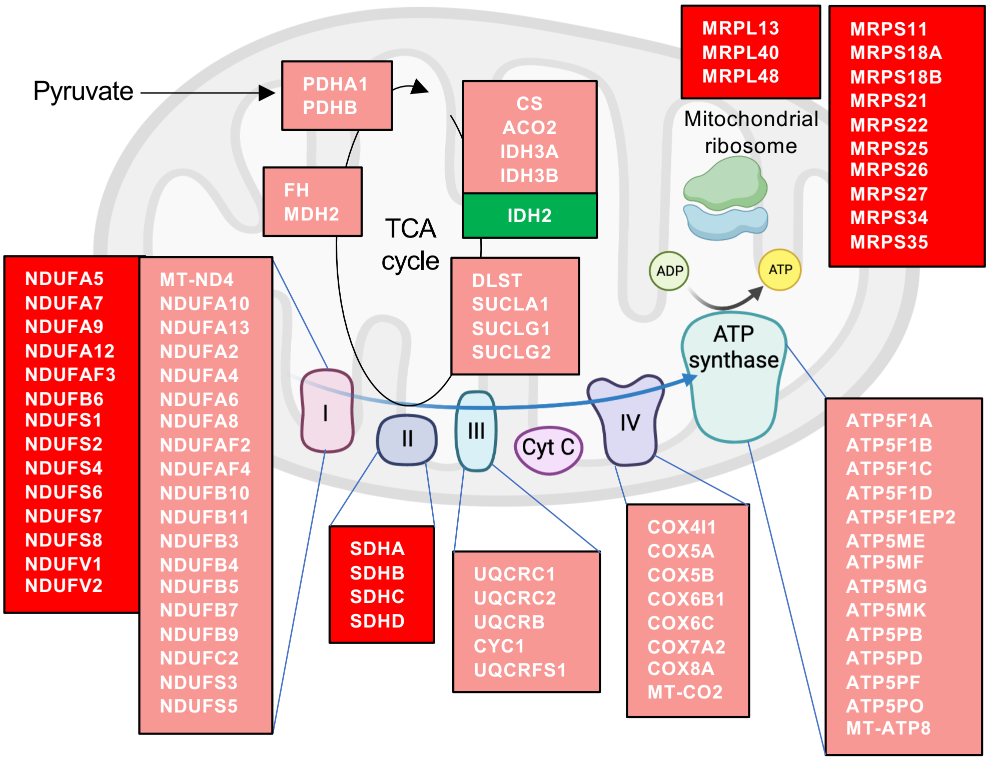

B

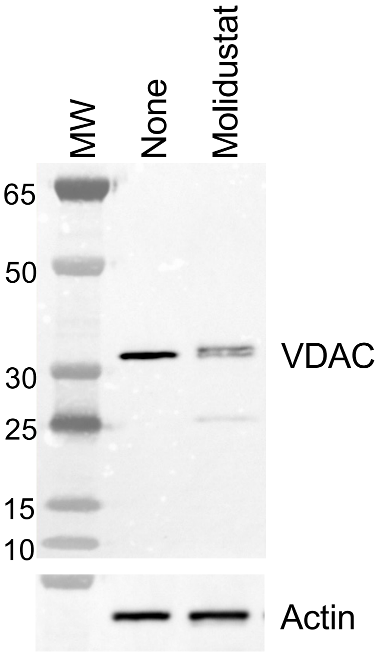

Supplementary Fig. 4 Downregulated mitochondrial proteins in Molidustat-treated Huh7 cell. (A) Mitochondrial proteins that were found to be downregulated in the proteomic analysis are shown depending on fold change in red ($Log_2(FC)<-1$; $p<0.05$) or pink ($Log_2(FC)<0$; $p<0.05$). IDH2 that is induced is shown in green. Created with BioRender.com. (B) Western-blot showing VDAC cleavage in Huh7 cells treated with Molidustat for 48 h. Actin level is used as loading control.

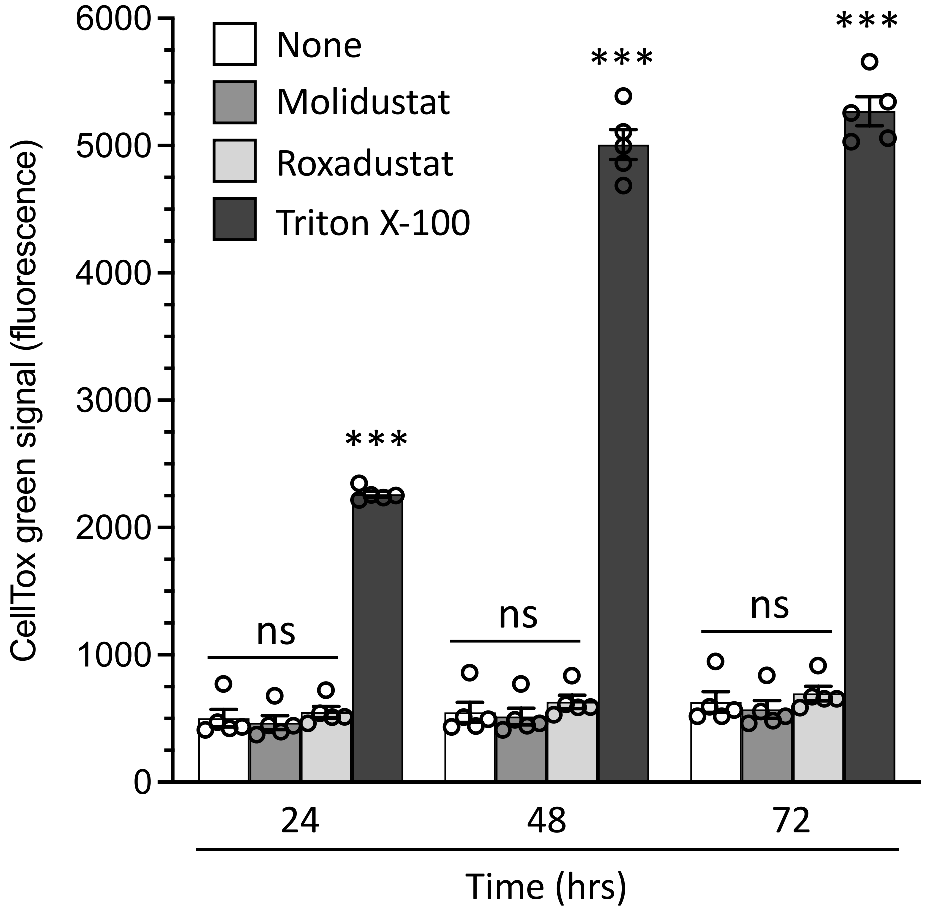

Supplementary Fig. 5 Molidustat and Roxadustat are not cytotoxic in Huh7 cell cultures. Cytotoxicity was evaluated by CellTox™ Green cytotoxicity assay. Huh7 were treated with DMSO alone, Molidustat (25 µM) or Roxadustat (25 µM) and cultured in the presence of the CellTox green dye. Fluorescence was measured every 24 h using TECAN M200 microplate reader. Treatment with 10% Triton X-100 one hour before measurement was used as cytotoxicity control.

Supplementary Fig. 6 Differential metabolites in cells treated or not by IPPA17-A04 or BAY-2402234. Changes in metabolite concentration are presented in log2 values of fold change (FC) calculated on the relative concentrations between treated vs control groups. P-values are calculated with a paired t-test. The list of differential metabolites is indicated (p-value<0.05 and log2|FC|>0.5 for at least one comparison). Decreased metabolites are colored in red and increased metabolites in green.

	IPPA17-A04		BAY-2402234	
Name	Log2 FC	P-value	Log2 FC	P-value
UDP	-5,29	0,004	-5,13	0,001
CTP	-4,63	0,000	-4,42	0,000
CDP	-4,44	0,001	-4,38	0,003
Uridine	-3,87	0,005	-2,74	0,009
N-Acetyl-aspartic acid	-2,96	0,000	-2,87	0,000
CDP-choline	-2,32	0,035	-1,45	0,003
N-Acetyl-glucosamine	-2,29	0,001	-2,70	0,002
Serine phosphate	-1,94	0,008	-1,60	0,012
2-Aminoadipic acid	-1,83	0,006	-2,08	0,005
6-Phosphogloconic acid	-1,63	0,054	-1,98	0,036
Cytidine	-1,60	0,001	-1,85	0,001
Isovalerylcarnitine	-1,02	0,001	-0,99	0,000
Cystathionine	-0,97	0,060	-1,15	0,029
N-Acetyl-methionine	-0,96	0,002	-1,14	0,003
2′-Deoxycytidine	-0,87	0,004	-0,64	0,020
Propionylcarnitine	-0,85	0,003	-0,76	0,007
GTP	-0,68	0,206	-1,53	0,017
N-Acetyl-glutamine	-0,68	0,003	-0,69	0,012
Glucosamine-6-phosphate	-0,65	0,001	-2,64	0,037
CMP	-0,63	0,004	-0,50	0,006
2-Oxoglutaric acid	-0,60	0,142	-0,83	0,028
Glutamic acid	-0,60	0,012	-1,38	0,031
Spermine	-0,56	0,007	-0,90	0,000
Glutathione	-0,55	0,005	-1,04	0,001
7-Methylguanine	-0,50	0,005	-1,61	0,000
2-Methylmalonic acid	-0,47	0,125	-0,95	0,016
Citric acid	-0,43	0,008	-1,04	0,003
2-Phosphoglyceric acid	-0,33	0,400	-0,81	0,039
Pyridoxal 5-phosphate	-0,14	0,047	-0,66	0,001
NAD+	-0,07	0,436	0,52	0,013
Inosine	-0,02	0,812	-2,00	0,001
Adenosine	0,03	0,727	-1,58	0,001
Maltotriose	0,08	0,454	-0,54	0,049
Guanosine	0,20	0,095	-1,24	0,002
NADH	0,21	0,617	1,36	0,011
AMP	0,32	0,187	1,41	0,014
N-Acetylneuraminic acid	0,37	0,251	1,35	0,019
GMP	0,38	0,132	1,43	0,017
Muramic acid	0,41	0,356	2,62	0,002
Adenine	0,52	0,005	1,13	0,005
Choline	0,56	0,014	0,35	0,027
Acetylhistidine	0,61	0,002	0,30	0,074
sn-glycero-3-Phosphocholine	0,64	0,017	-0,08	0,611
Taurochenodeoxycholic acid/Taurodeoxycholic acid	0,84	0,000	-0,01	0,900
Creatine phosphate	1,33	0,036	1,42	0,008
Malic acid	1,56	0,005	1,38	0,009
Acetylcarnitine	1,64	0,000	1,63	0,000
Isocitric acid	1,76	0,011	1,65	0,025
Ascorbic acid	2,01	0,016	1,38	0,058
Hypoxanthine	5,95	0,000	6,31	0,000
Carbamoylaspartate	7,68	0,000	7,68	0,000
Dihydroorotic acid	8,17	0,000	8,09	0,000

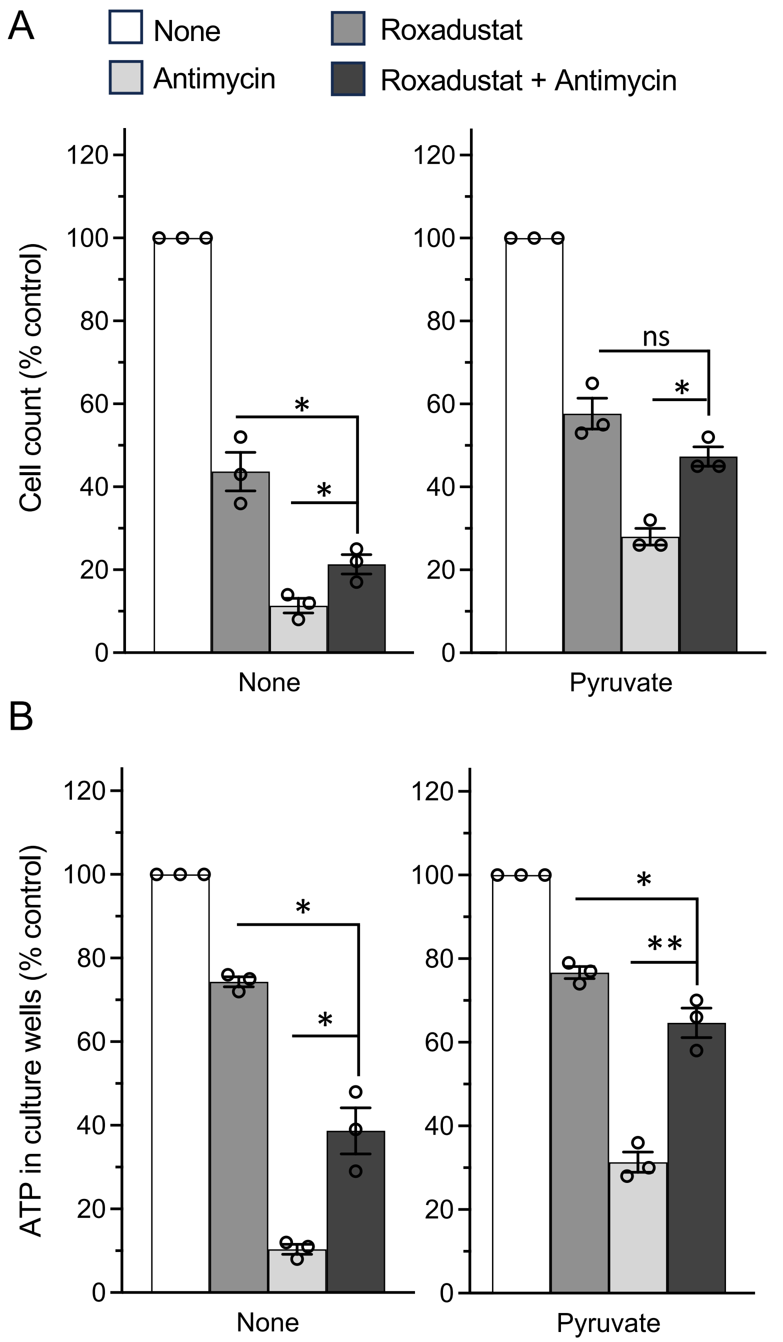

Supplementary Fig. 7 Proliferation of Huh7 cells is inhibited by Complex III inhibitor antimycin, but is restored in the presence of pyruvate and Roxadustat. (A) Huh7 cells were treated with DMSO alone, Roxadustat (25 µM) and antimycin (1 µM) in the absence or presence of pyruvate (1 mM). After 72 h, cell counts were determined by Hoechst staining and quantification of the fluorescence signal (A) and ATP level in culture wells that reflects the number of viable cells was measured (B). Data were normalized to untreated control (None) without (left panels) or with pyruvate (right panels). Means ± SEM of three experiments in triplicate.

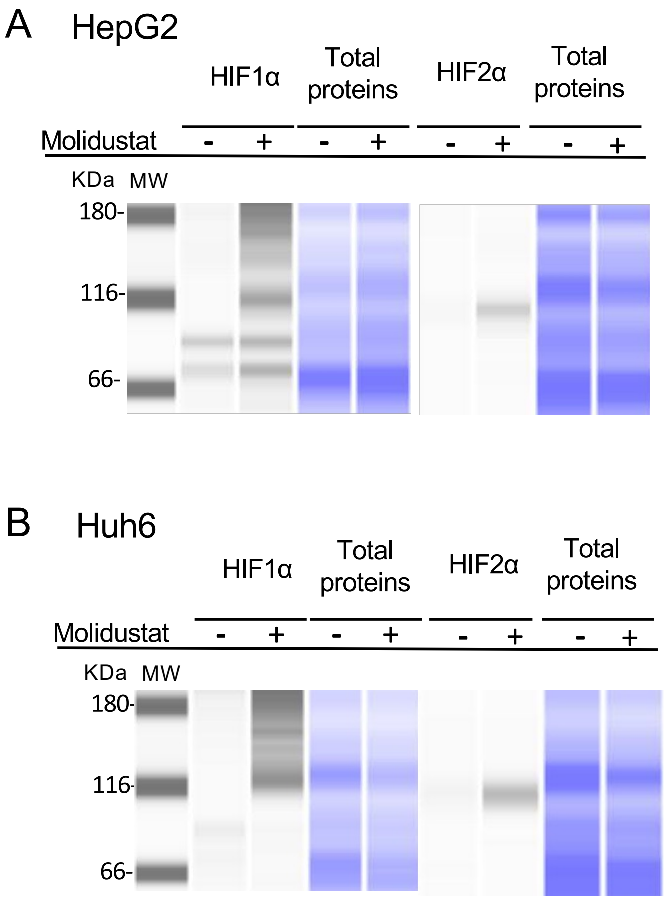

Supplementary Fig. 8 (A-B) HepG2 (A) or Huh6 (B) cells were treated for 24 h with Molidustat (25 µM) or DMSO alone, and HIF-1α and HIF-2α expression were determined by Jess analysis. Total proteins loaded in the capillaries are presented on the right panels.
